# Light as a Weapon: Redefining Cancer Care with Photodynamic Therapy

**DOI:** 10.7150/ijbs.125584

**Published:** 2026-01-14

**Authors:** Kulsoom X, Wajahat Ali, Yue Chong, Zhenlong Wang, Li Xue, Fu Wang

**Affiliations:** 1 Department of Urology, The Second Affiliated Hospital of Xi'an Jiaotong University, Xi'an, Shaanxi 710004, China; 2 Institute of Medical Engineering, School of Basic Medical Sciences, Xi'an Jiaotong University, Xi'an 710061, China.

**Keywords:** photodynamic therapy (PDT), cancer treatment, photosensitizers, nanocarriers, immunotherapy, combination therapy

## Abstract

Photodynamic therapy (PDT) has evolved over the past decade into a versatile cancer treatment modality, fueled by advances in photosensitizer design, nanotechnology, and immunotherapy. Smarter photosensitizers, ranging from activatable and oxygen-independent scaffolds to red/NIR-absorbing chromophores, have expanded tumor selectivity and tissue penetration. Nanocarrier-based delivery systems have improved solubility, biodistribution, and combinatorial theranostic potential, while innovative light sources such as upconversion nanoparticles, implantable LEDs, and X-ray-driven PDT are addressing penetration constraints. Importantly, PDT is no longer confined to local cytotoxicity but is increasingly recognized as an immunomodulatory platform capable of synergizing with immune checkpoint blockade (ICB) and *in situ* vaccination strategies. However, despite this progress, significant barriers remain, including poor standardization of dosimetry, heterogeneity in tumor hypoxia, unresolved safety concerns regarding nanocarriers, and limited clinical validation of combination regimens. This highlight review critically evaluates these advances and bottlenecks, outlining how PDT can realistically transition from a niche light-based therapy into a central component of precision oncology and next-generation immunotherapy.

## 1. Introduction

Over the past decade, PDT has emerged as one of the most versatile and promising modalities in cancer treatment, owing to its unique mechanism of action, spatiotemporal selectivity, and minimal systemic toxicity 1. Unlike conventional therapies such as chemotherapy and radiotherapy, PDT utilizes light, a photosensitizer, and molecular oxygen to generate cytotoxic reactive oxygen species (ROS), thereby inducing tumor cell death, disrupting tumor vasculature, and stimulating anti-tumor immunity 2, 3. These multifaceted effects position PDT not only as a direct cytotoxic intervention but also as an immune-modulatory platform capable of synergizing with next-generation cancer therapies 4, 5. This evolution is based on three central pillars: the refinement of photosensitizers (PSs), the development of nanotechnology-mediated delivery systems, and synergistic interactions with immunotherapy 6, 7. Similarly, the advent of advanced architectures such as NIR-responsive lanthanide@MOF nanocatalysts and X-ray-induced PDT systems brings the promise of treating deep-seated tumors under previously prohibitive light-penetration constraints 8, 9.

Over the last decade, significant progress has been made in refining PDT for clinical translation. Advances in photosensitizer design, including the development of second and third-generation agents with improved photophysical properties, have significantly enhanced tumor specificity and therapeutic outcomes 10. Simultaneously, the integration of nanotechnology has revolutionized drug delivery, enabling enhanced permeability and retention (EPR)-driven tumor accumulation, targeted delivery, and stimulus-responsive activation 11. Parallel to these chemical and material innovations, the engineering of advanced light-delivery systems, such as implantable optical fibers, upconversion nanoparticles, and X-ray-activated scintillators, has expanded the applicability of PDT to deep-seated and otherwise intractable tumors 12.

Furthermore, growing evidence highlights PDT's ability to induce immunogenic cell death (ICD), leading to systemic anti-tumor immune responses 1. This has catalyzed a paradigm shift in PDT research, transforming it from a localized treatment into a strategic ally of immunotherapies, including immune checkpoint inhibitors and cancer vaccines 13. The past decade has thus witnessed the evolution of PDT from a niche, light-dependent cytotoxic therapy into a broad-spectrum, multifunctional platform at the interface of oncology, immunology, and nanomedicine 14.

Despite these remarkable advances, the clinical trajectory of PDT is still constrained by several unresolved challenges. Light penetration into deep tissues remains inconsistent, tumor hypoxia continues to blunt therapeutic efficacy, and the safety and scalability of nanocarrier systems have yet to be validated in large-scale studies. Furthermore, while PDT-induced ICD has opened the door for immunotherapy combinations, most evidence remains preclinical, with limited translation into standardized clinical protocols. These controversies ranging from dosimetry optimization to the reproducibility of multifunctional nanoplatforms highlight the urgent need for a critical synthesis that goes beyond summarizing technological progress.

Accordingly, this highlight review provides a focused and critical evaluation of the innovations in PDT over the past decade. We emphasize (1) the emergence of advanced photosensitizers and delivery strategies, (2) novel light-activation methodologies that extend treatment to previously inaccessible tumors, and (3) the immunological synergies achieved when PDT is combined with immunotherapy. By balancing progress with translational bottlenecks, this review aims to define the realistic opportunities and remaining challenges that must be addressed for PDT to transition from experimental promise to a central pillar of precision oncology (Table 1).

### 1.1. Mechanistic Basis of Photodynamic Therapy

**Figure 1:**
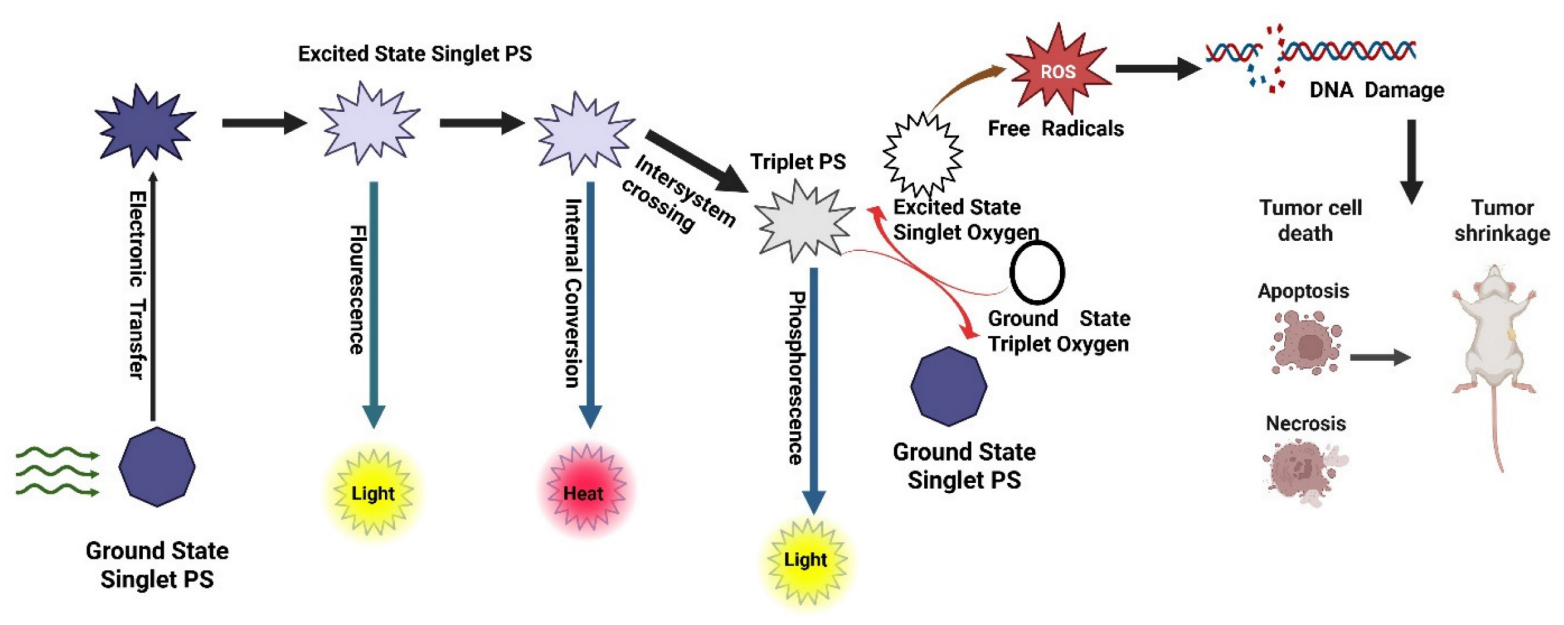
Mechanism of photodynamic therapy for the treatment of cancer.

PDT is a light-activated therapeutic modality that destroys malignant or infected tissues by spatiotemporally controlling the generation of ROS. The overall mechanism couples three non-toxic components a PS, molecular oxygen, and light of an appropriate wavelength to trigger photochemical and biological events that culminate in cell death and immune activation (Figure 1) 15.

#### 1.1.1. Photophysical Excitation and Energy Transfer

Upon illumination, a PS absorbs photons and is promoted from its ground singlet state (S₀) to an excited singlet state (S₁). This short-lived S₁ state can either fluoresce or undergo intersystem crossing (ISC) to a long-lived triplet state (T₁), whose lifetime (10⁻⁶-10⁻³ s) enables diffusion and interaction with biomolecules or oxygen 10. The quantum yield of ISC and the extinction coefficient determine the photochemical efficiency of a PS. Molecular engineering such as heavy-atom substitution, donor-acceptor conjugation, and π-extension has been used to improve ISC and red-shift absorption into the NIR window for deeper tissue penetration 16.

#### 1.1.2. Type I and Type II Photochemical Pathways

The excited triplet PS can react by two primary routes:

- Type II energy-transfer reaction: The PS transfers its triplet energy to ground-state molecular oxygen (³O₂), producing singlet oxygen (¹O₂) the hallmark cytotoxic species in classical PDT. ¹O₂ rapidly oxidizes unsaturated lipids, amino acids, and nucleic acids within a diffusion radius of ~20 nm, leading to irreversible biomolecular damage 17.
- Type I electron- or hydrogen-transfer reaction: The triplet PS reacts with biomolecules to generate radical species such as superoxide (O₂•⁻), hydroxyl radicals (•OH), and hydrogen peroxide (H₂O₂). These secondary ROS cause sustained oxidative stress and mitochondrial dysfunction. Importantly, Type I mechanisms dominate under hypoxic conditions, a frequent feature of solid tumors 18. Recent research emphasizes Type I-dominant or oxygen-independent PDT, utilizing platinum(IV), aza-BODIPY, and quinone-based PSs capable of direct photooxidation without molecular oxygen, thereby overcoming hypoxia-associated resistance 19.

#### 1.1.3. Subcellular Localization and Pathways of Cell Death

The biological outcome of PDT is dictated by where the PS resides within cells.

- Mitochondrial PSs (TSPO-targeted porphyrins) disrupt the mitochondrial membrane potential, release cytochrome c, and activate the caspase cascade, culminating in apoptosis 20.
- Lysosomal localization provokes membrane permeabilization, protease release, and necrotic cell death.
- Endoplasmic reticulum (ER)-localized PSs initiate unfolded-protein response and Ca²⁺ leakage, leading to ER-stress-mediated apoptosis and strong immunogenic signaling. Subcellular targeting is thus a rational design parameter to control therapeutic outcomes 20.

#### 1.1.4. Molecular and Cellular Consequences

The immediate result of ROS attack is **oxidative modification of lipids, proteins, and nucleic acids**, disrupting membrane integrity and signaling pathways. At the cellular level, PDT triggers:

- **Apoptosis**, via mitochondrial and caspase-dependent pathways.

**Necrosis**, when ROS burden exceeds cellular antioxidant capacity.

- **Autophagy**, a protective or lethal response depending on context. Oxidative stress also causes **DNA double-strand breaks** and lipid peroxidation, amplifying cytotoxicity. Recent omics studies reveal up-regulation of p53, HSP70, and Mitogen-Activated Protein Kinase (MAPK) pathways after PDT exposure, correlating ROS levels with gene expression remodeling 21.

#### 1.1.5. Immunogenic Cell Death and Systemic Antitumor Immunity

Beyond direct tumor ablation, PDT acts as a potent *in situ* cancer vaccine. ROS-induced stress exposes damage-associated molecular patterns (DAMPs) calreticulin exposure, ATP secretion, and HMGB1 release that recruit and activate dendritic cells (DCs). Activated DCs present tumor antigens to T cells, generating durable cytotoxic immunity 16. PDT-induced DNA damage further activates the cGAS-STING pathway, leading to type I interferon production and enhanced responsiveness to ICB. These findings underpin modern photo-immunotherapy and the combination of PDT with PD-1/PD-L1 or STING agonists.

#### 1.1.6. Oxygen Modulation and Self-Sustaining ROS Generation

Hypoxia remains a critical limitation of classical PDT. To counter this, oxygen-generating nanoplatforms incorporating MnO₂, catalase, or perfluorocarbon components decompose endogenous H₂O₂ or deliver dissolved O₂, thereby sustaining ROS generation in poorly perfused tumors 3. Additionally, Type I-dominant and two-photon PSs enable deeper tissue treatment by leveraging high-energy NIR photons or oxygen-independent mechanisms.

## 2. Key Advances in the Past Decade

Over the last decade, PDT has been revolutionized by the emergence of activatable photosensitizers that respond selectively to tumor-specific cues such as hypoxia or acidic pH, thereby improving therapeutic precision while reducing systemic toxicity 22. Parallel advances in nanocarrier delivery systems, including MnO₂-based nanoplatforms that alleviate tumor hypoxia and facilitate combined PDT-PTT, have yielded superior tumor suppression in preclinical models 23. To address light penetration barriers, UCNPs have been employed to convert near-infrared (NIR) excitation into visible wavelengths, enabling effective PDT in deep-seated tumors 15. Synergistic combination therapies, such as PDT coupled with ICB or photothermal therapy, have shown significant promise in enhancing systemic antitumor immunity and durable responses 24. Together, these breakthroughs highlight PDT's transition from a localized phototoxic intervention into a multifunctional, immune-potentiating platform poised for clinical translation. Recent PDT innovations are not merely incremental but strategically engineered to overcome historical limitations that have constrained clinical translation. For example, bioluminescent PDT platforms directly bypass the classical problem of shallow tissue penetration by replacing external light sources with internal photon generation, thereby addressing a challenge that conventional NIR-PDT and upconversion systems could not fully solve.

### 2.1. Advanced Photosensitizers

Recent advancements in PDT for cancer treatment have focused on developing smarter photosensitizers that enhance the precision and efficacy of the treatment while minimizing adverse effects 25. PDT is a minimally invasive cancer treatment that utilizes photosensitizers activated by specific wavelengths of light to generate ROS, resulting in the death of cancer cells and tumor destruction 26.

One of the significant developments in enhancing the selectivity and effectiveness of photosensitizers is the integration of advanced molecular designs. For instance, synthetic glycosylated photosensitizers are being developed to improve solubility, stability, and tumor specificity, addressing challenges such as skin photosensitivity and inefficacy under hypoxic conditions 27. Glycosylation can significantly enhance the targeting ability of photosensitizers and improve the overall effectiveness of PDT.

The development of photosensitizers with bioorthogonal delivery systems is also noteworthy. These systems allow targeted deployment and activation of photosensitizers within specific tumor tissues, thereby improving precision and treatment outcomes 28. Additionally, phototheranostic agents that combine optical imaging, targeted delivery, and photodynamic treatment enhance the detection and eradication of malignant cells with higher localization and reduced side effects 28.

#### 2.1.1 Activatable Photosensitizers

O'Mahoney *et al.* explored how fluorescence and thermal imaging can be used to monitor non-melanoma skin cancers during PDT, by simultaneously assessing protoporphyrin IX (PpIX) fluorescence (as a marker of photosensitizer accumulation) and skin surface temperature (as an indicator of metabolic and vascular activity). Results showed that trunk lesions displayed significantly higher baseline fluorescence and temperatures compared with lesions on the lower legs, correlating with greater PpIX accumulation and potentially improved PDT sensitivity. Interestingly, patients experienced more pain during treatment of extremity lesions, despite their lower fluorescence and temperature values. Notably, the overall tumor clearance rate remained high (~80% at 12 months), confirming the effectiveness of PDT but highlighting site-specific physiological differences that may influence photosensitizer uptake, thermal response, and patient experience 29. Fluorescence and thermal imaging provide valuable, non-invasive biomarkers for predicting PDT response, optimizing light dosimetry, and improving personalized treatment strategies for skin cancers. While the study nicely shows site-specific variability in PpIX accumulation and thermal response, it does not directly establish whether these imaging readouts can prospectively predict patient outcomes. Larger, multicentric validation is required to position fluorescence/thermal imaging as a clinical biomarker. This study addresses the major challenge of unpredictable photosensitizer accumulation in clinical PDT. By correlating fluorescence with thermal patterns, the work provides a non-invasive method to predict lesion responsiveness before irradiation something conventional PDT could not achieve. However, the study does not establish whether these imaging metrics can consistently forecast clinical outcomes, and large-scale validation is still required.

**Figure 2:**
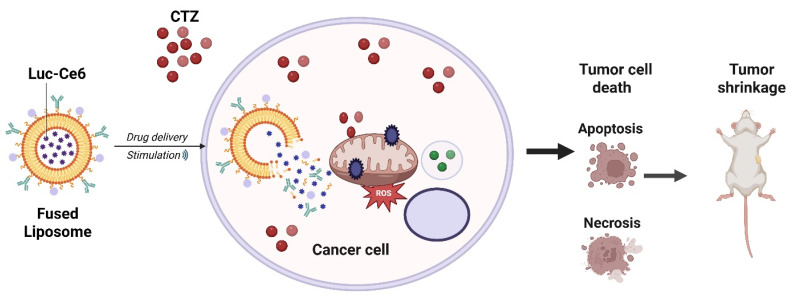
Bioluminescent photodynamic therapy (BL-PDT) strategy using natural-molecule systems. Internal bioluminescence activates the photosensitizer without external light, overcoming tissue penetration limits and enabling complete tumor regression while preventing metastasis.

Yan *et al.* reported the development of an all-natural molecule-based bioluminescent PDT (BL-PDT) platform capable of achieving complete tumor regression while preventing metastatic spread. Unlike conventional PDT, which requires external light irradiation, this system employs a bioluminescent reaction as an internal light source to activate the photosensitizer, thereby overcoming the major limitation of limited light penetration in deep-seated tumors. Preclinical models demonstrated that the natural-molecule-based BL-PDT induced robust generation of ROS, leading to extensive tumor necrosis, vascular disruption, and immune activation. Strikingly, treatment not only caused complete regression of primary tumors but also suppressed distant metastasis, highlighting its systemic therapeutic potential. The safety profile was favorable, with minimal off-target toxicity and excellent biocompatibility, underscoring its translational promise (Figure 2) 30. This work directly solves the classical limitation of shallow light penetration by generating internal bioluminescence that activates the photosensitizer without external light. This represents a major conceptual innovation over NIR- or UCNP-based methods, which still depend on external excitation. Remaining limitations include low bioluminescence intensity and the difficulty of sustaining enzyme-driven reactions *in vivo*. Overall, the work establishes bioluminescent PDT using natural molecules as a breakthrough strategy that bypasses external light dependency, enhances treatment depth, and delivers potent anti-metastatic efficacy representing a significant step toward next-generation cancer theranostics. This approach elegantly bypasses the light penetration barrier; however, the biochemical complexity of sustaining bioluminescence *in vivo*, the potential immunogenicity of enzymes, and the energy yield remain bottlenecks. Translation will require standardized bioluminescent reactions compatible with human physiology.

#### 2.1.2 Red/NIR-Absorbing Photosensitizers

**Figure 3:**
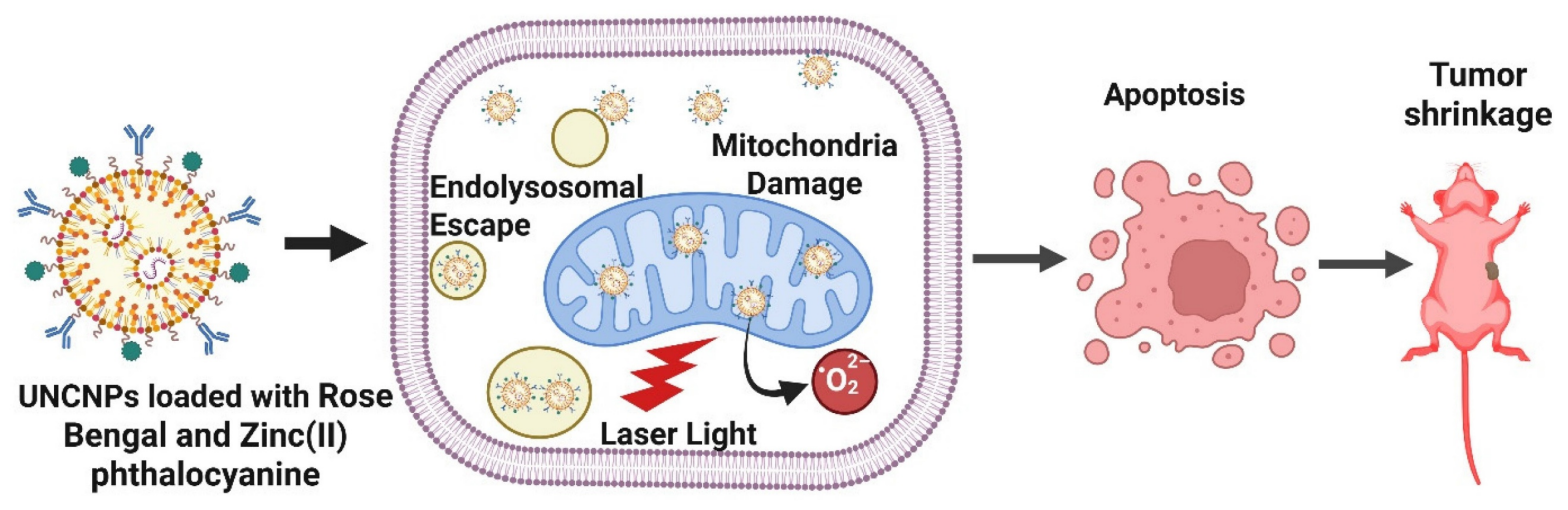
Dual-loaded upconversion nanoparticles (Rose Bengal + Zn(II) phthalocyanine) for organelle-targeted PDT. Dynamic trafficking from endosomes to mitochondria enhances ROS production and induces mitochondria-mediated apoptosis, demonstrating the role of subcellular localization in PDT efficacy.

Dual-loaded upconversion nanoparticles carrying Rose Bengal and Zinc(II) phthalocyanine enabled real-time subcellular tracing and spatiotemporal control of PDT. The nanophotosensitizers dynamically trafficked from endosomes to mitochondria, where their accumulation triggered potent ROS generation and mitochondria-mediated apoptosis. This study demonstrates how organelle-level targeting and dynamic localization significantly enhance the precision and therapeutic efficacy of PDT, offering a powerful platform for next-generation nanomedicine. Chang *et al.* developed innovative poly(allylamine)-modified UCNPs dual-loaded with two photosensitizers Rose Bengal and Zinc(II) phthalocyanine and activated by NIR light. These nanophotosensitizers are engineered to accumulate in specific subcellular organelles, enabling precise spatiotemporal control over PDT at the organelle level. Upon cellular uptake, the UCNPs were observed to first reside in endosomes/lysosomes, later migrating to the cytoplasm and ultimately accumulating in the mitochondria. The most potent apoptotic effect occurred when these nanophotosensitizers localized within mitochondria, confirming that subcellular positioning directly influences the efficacy of PDT (Figure 3). The resulting cell death followed a mitochondria-mediated apoptotic pathway, underscoring the importance of organelle-targeted delivery and precise timing in enhancing PDT outcomes 31. The study overcomes the problem of non-specific subcellular localization by demonstrating controlled trafficking from endosomes to mitochondria, where ROS are maximally effective. This provides a mechanistic solution to earlier first-generation nanoparticles that accumulated randomly in the cytosol. Nonetheless, quantum yield and *in vivo* penetration efficiency remain hurdles for clinical deployment. Insights into organelle trafficking are strong, but the quantum yield and phototoxicity thresholds for *in vivo* deep tumors remain unclear. The challenge lies in balancing emission efficiency with long-term biocompatibility.

**Figure 4:**
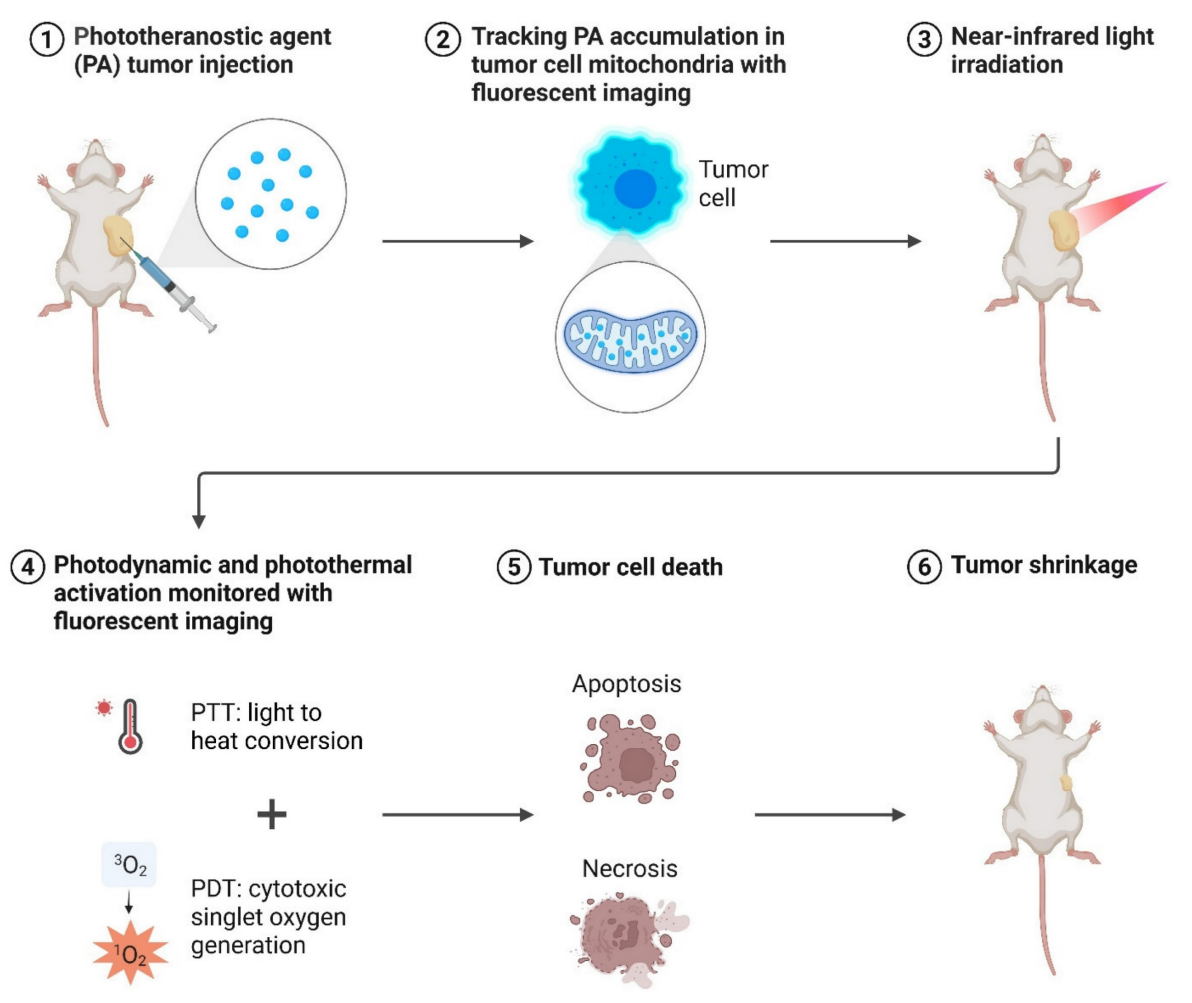
Raman/fluorescence dual-modal imaging nanoplatform for precision cancer therapy. A plasmonic gold core enables Raman-based tumor margin detection, while surface photosensitizers provide fluorescence-guided PDT and PTT synergy. The system achieved effective tumor ablation with minimal recurrence.

Fu *et al.* designed a Raman/fluorescence (R/F) dual-modal imaging nanoplatform that integrates precise tumor diagnosis with synergistic photothermal therapy (PTT) and PDT. The nanoplatform was engineered using a core-shell nanostructure: a plasmonic gold core provided strong Raman scattering for high-sensitivity detection. At the same time, the outer layer was functionalized with a photosensitizer and fluorescent probe for complementary fluorescence imaging and PDT. The nanoplatform enabled both high-resolution Raman imaging (for precise identification of tumor boundaries) and real-time fluorescence imaging (for intraoperative guidance), ensuring accurate tumor localization. Upon NIR laser irradiation, the platform induced a combined photothermal and photodynamic effect, leading to enhanced cancer cell killing compared to either modality alone. Cancer cells treated with the nanoplatform showed significant apoptosis and necrosis under dual-mode irradiation, with survival rates dropping much lower than single-mode treatments. The nanoplatform achieved effective tumor ablation with minimal recurrence, while also allowing non-invasive real-time monitoring of treatment progress (Figure 4). Importantly, no apparent systemic toxicity was observed, indicating good biocompatibility 32. This work solves two major challenges simultaneously: (1) inaccurate tumor margin detection and (2) insufficient therapeutic depth. The Raman core allows high-resolution tumor identification, while the integrated PS enables dual PDT/PTT treatment. Remaining barriers include scalability of plasmonic nanostructures and long-term metal clearance. This work highlights a precision theranostic approach where Raman/fluorescence dual-modal imaging provides accurate tumor visualization and guides synergistic PTT/PDT for efficient and safe cancer therapy. The integration of diagnosis and treatment into a single nanoplatform demonstrates significant potential for clinical translation in precision oncology. The dual imaging and therapy paradigm is powerful for precision oncology; yet, scaling plasmonic core-shell systems and ensuring long-term clearance remain key translational barriers.

**Figure 5:**
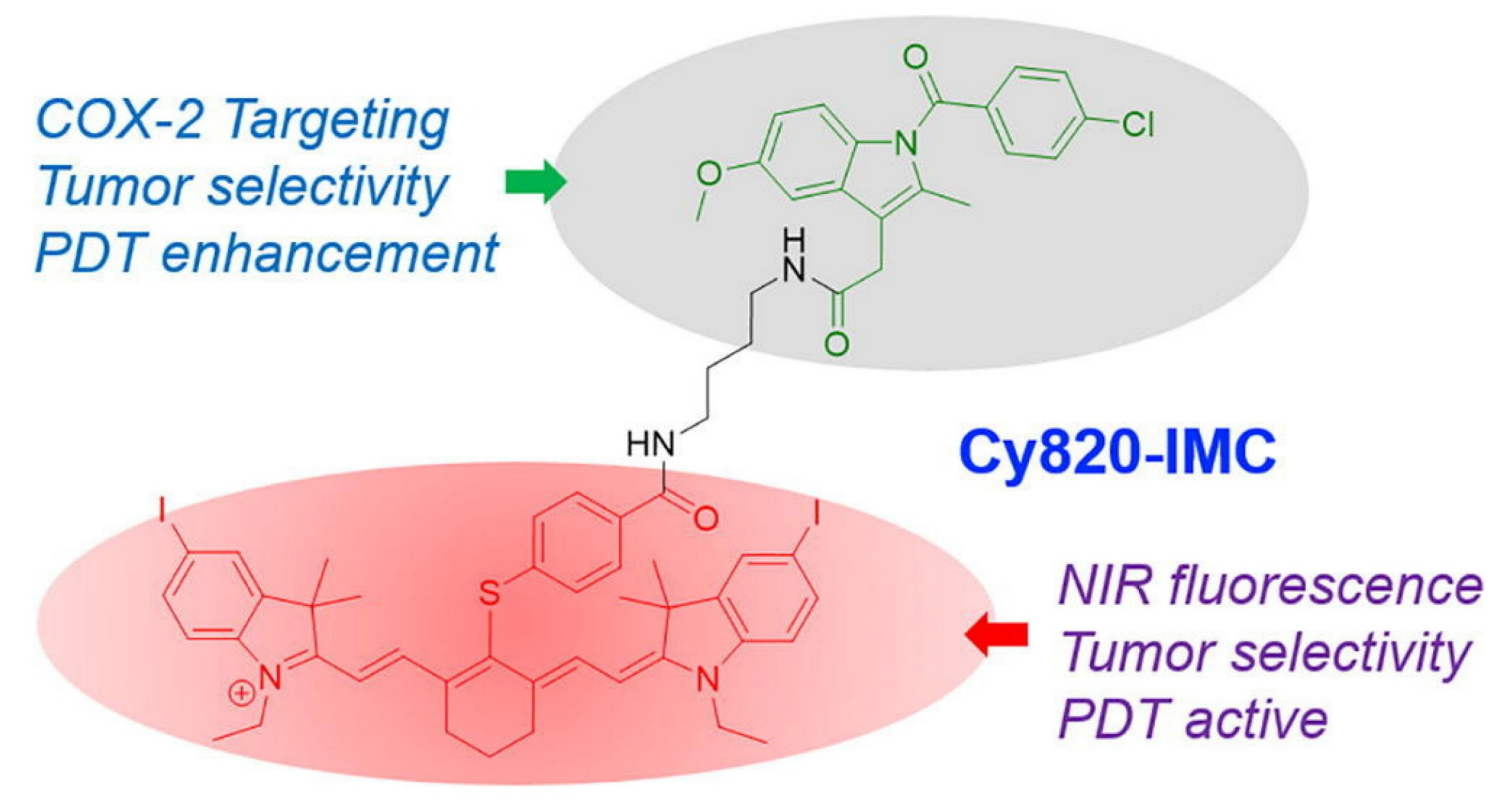
Indomethacin-conjugated NIR photosensitizer (Cy820-IMC). The dual-purpose design integrates COX-2 targeting with NIR light activation, producing synergistic anti-inflammatory and photodynamic effects with deep tissue penetration and improved tumor selectivity 33. The figure was adapted from *Siriwibool, S., et al., 2022* with permission from Elsevier.

An innovative strategy in PDT involves the development of indomethacin-based photosensitizers designed to operate in the NIR region. Unlike conventional PDT agents that suffer from limited tissue penetration and poor tumor selectivity, this approach leverages the anti-inflammatory drug indomethacin as a molecular scaffold, integrating it with NIR-absorbing chromophores to generate a dual-functional therapeutic agent. Siriwibool *et al.* designed a novel photosensitizer, Cy820-IMC, by conjugating the NIR heptamethine cyanine dye Cy820 with indomethacin, a COX-2-targeting non-steroidal anti-inflammatory drug (NSAID). This dual-purpose design aims to enhance the selective delivery and efficacy of PDT in cancer cells. The indomethacin conjugation not only enables selective tumor targeting through cyclooxygenase-2 (COX-2) overexpression but also provides synergistic anti-inflammatory and anti-tumor effects. Upon NIR light irradiation, the conjugate efficiently produces ROS, leading to potent phototoxicity, enhanced apoptosis, and suppression of tumor progression (Figure 5). The deeper tissue penetration of NIR wavelengths further enhances therapeutic efficacy against solid tumors while minimizing collateral damage to healthy tissues. Cy820-IMC demonstrates superior tumor targetability and photodynamic effectiveness, offering a promising scaffold for further development into clinically viable NIR-activated sensitizers 33. This strategy solves the dual challenge of poor tumor selectivity and inadequate NIR absorption. By repurposing the COX-2-targeting NSAID indomethacin, tumor-specific accumulation significantly improves. The NIR window further enhances depth of treatment. A limitation is the dependence on COX-2 overexpression, which varies among tumor types. This dual-targeted, drug-repurposed design highlights a promising avenue for next-generation PDT, combining precision cancer targeting, improved light penetration, and synergistic pharmacological effects.

#### 2.1.3 Hypoxia-Compatible / Oxygen-Independent Photosensitizers

**Figure 6:**
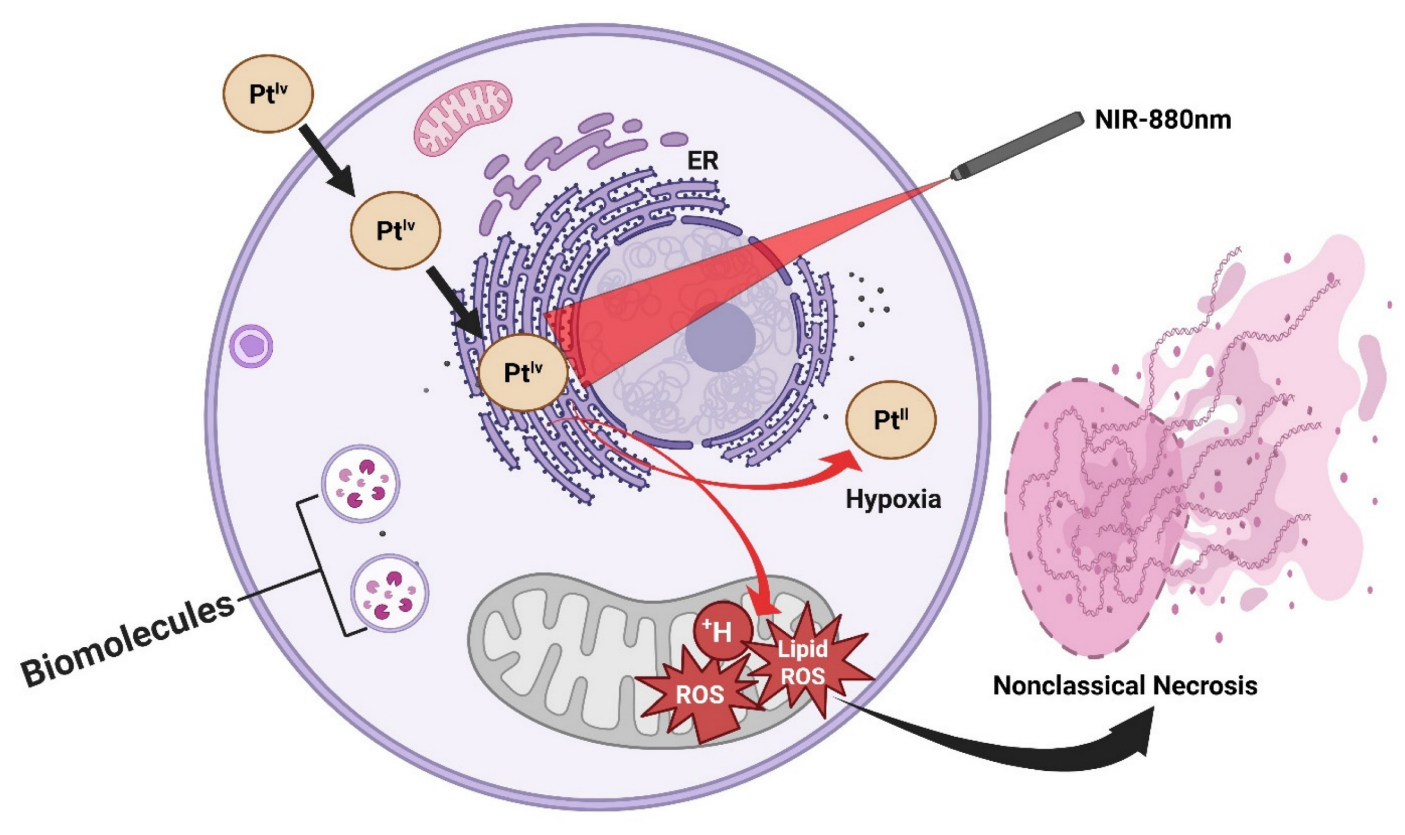
Oxygen-independent NIR-activated Pt(IV) photosensitizers. These agents bypass tumor hypoxia by directly photooxidizing biomolecules, generating cytotoxic platinum(II) species even under oxygen-deficient conditions, thus broadening PDT applicability to hypoxia-resistant tumors.

A longstanding limitation of conventional PDT lies in its strict dependence on molecular oxygen, as the cytotoxic effects are primarily mediated through the generation of ROS (¹O₂). This severely constrains PDT efficacy in hypoxic tumors, such as prostate and other solid malignancies, where oxygen partial pressure is inherently low. Recent work has addressed this challenge through the design of NIR-activated platinum(IV) complexes that circumvent oxygen dependency by directly photooxidizing critical biomolecules. These platinum(IV) prodrugs are constructed with NIR-absorbing ligands that extend light activation into the biologically transparent NIR window (650-850 nm), allowing deeper tissue penetration compared to visible-light systems. Upon irradiation, the complexes undergo photoexcitation, generating highly reactive platinum(II) species that directly oxidize DNA bases, amino acid residues, and lipid membranes, without the requirement for oxygen. This oxygen-independent photochemistry not only sustains cytotoxic action in hypoxic microenvironments but also bypasses tumor defense mechanisms associated with hypoxia-induced survival signaling (Figure 6). *In vitro* and *in vivo* studies have demonstrated potent anticancer activity under hypoxic conditions, with significant tumor growth inhibition achieved in xenograft models using clinically relevant light doses. Notably, the structural modularity of platinum(IV) scaffolds enables the tuning of both photoreactivity and pharmacokinetics, thereby raising the possibility of combining phototherapy with traditional platinum chemotherapy on a single platform 34. This class of NIR-activated platinum(IV) complexes represents a paradigm shift in photo-based cancer therapy: rather than amplifying oxygen or alleviating hypoxia, the therapeutic design strategically sidesteps oxygen altogether. Such agents hold promise for expanding photomedicine into hypoxia-resistant tumors, positioning them as complementary or even superior alternatives to traditional PDT in challenging clinical contexts. Represents a paradigm shift, but an entirely new mechanism demands deep toxicology before translation; the risk of off-target DNA damage is high. This study provides a direct solution to the hypoxia barrier, a central limitation in classical PDT. Instead of generating oxygen-dependent ROS, Pt(IV) complexes produce cytotoxic Pt(II) species independently of oxygen availability. This represents a paradigm shift; however, concerns remain regarding DNA damage to normal tissues and long-term toxicity.

**Figure 7:**
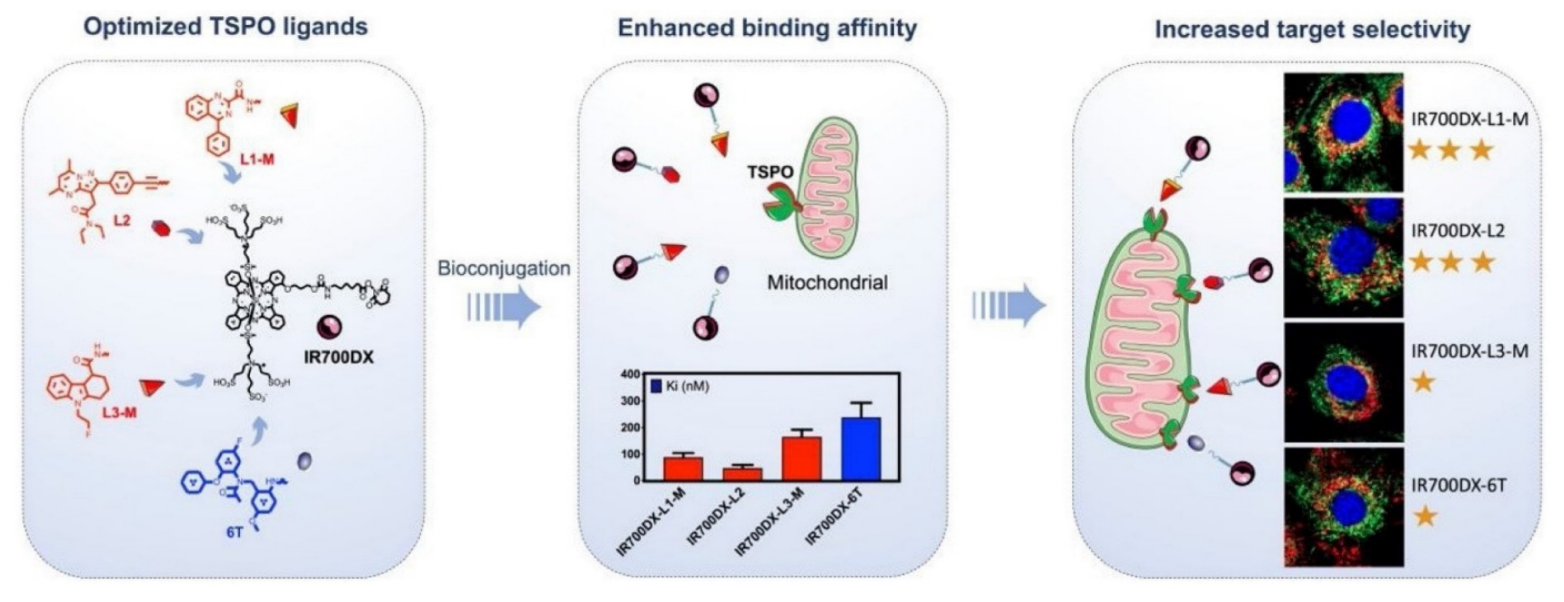
IR700DX-L1-M, IR700DX-L2, IR700DX-L3-M, and IR700DX-6T represent distinct TSPO-targeted photosensitizer conjugates. “L1-M,” “L2,” and “L3-M” correspond to optimized TSPO-binding ligands with different substituent patterns that modulate mitochondrial affinity and TSPO interaction strength. “6T” denotes a tricyclic TSPO-affinity scaffold with a distinct binding orientation compared to the L-series ligands. All constructs share the IR700DX photosensitizing core, but the structural differences in the appended TSPO ligands account for variations in binding affinity, mitochondrial localization, and target selectivity 20. The figure was adapted from *Xie, Q., et al., 2021* with permission from Elsevier.

Recent research has explored TSPO-targeted strategies to enhance the efficacy of PDT. This minimally invasive cancer treatment modality employs light-activated photosensitizers to induce ROS and trigger tumor cell death. The studies highlight that TSPO-targeted PDT can induce multiple cell death pathways, including apoptosis, autophagy, and necrosis, depending on the tumor microenvironment (TME) and the type of photosensitizer used. Xie *et al.* report that TSPO-targeted PDT induces rapid mitochondrial depolarization, cytochrome c release, and caspase activation, resulting in significant cancer cell killing at lower light doses. *In vitro* assays reveal improved selectivity with minimal toxicity to normal cells, while *in vivo* tumor models demonstrate greater tumor regression and a survival benefit compared to conventional PDT. Some studies have also noted the induction of autophagy and necrosis in resistant tumors, suggesting a multi-modal cell death mechanism (Figure 7) 20. TSPO-directed photosensitizers demonstrated superior tumor localization and phototoxicity, with quantitative results showing up to 2-3 fold higher ROS generation and enhanced tumor-to-normal tissue uptake ratios. These outcomes highlight the translational advantage of TSPO targeting, not only for improving PDT efficacy but also for potentially combining with immune-based therapies. TSPO-targeted PDT enhances mitochondrial damage, strengthens tumor selectivity, and achieves better therapeutic outcomes than standard PDT, underscoring its potential in precision cancer therapy. This work addresses the issue of poor tumor selectivity by exploiting TSPO overexpression in cancer mitochondria, allowing highly localized ROS production. This improves therapeutic precision compared with non-targeted PSs. The main limitation is variable TSPO expression across tumors, requiring biomarker-based patient selection. Still, clinical success depends on demonstrating TSPO overexpression as a robust and universal biomarker across patient populations.

### 2.2. Nanocarrier Delivery Systems

Nanotechnology plays a significant role in the enhancement of PDT through the use of nanoparticles that can deliver oxygen, enhance photosensitizer stability, and target tumors more effectively 35. These nanoparticles can carry multiple therapeutic agents, allowing for the co-delivery of drugs that enhance the efficacy of PDT. This approach has been successful in improving the uptake and localization of therapeutic agents at the tumor site, thus enhancing treatment outcomes. Furthermore, the use of nanotechnology in photosensitizers has shown promising results. Nanocarriers loaded with photosensitizers enhance solubility, optical absorption, and tumor-targeting capabilities, which are crucial for treating aggressive and radiation-resistant cancers, such as melanoma 36. Plasmonic nanoparticle-based hybrid photosensitizers also exhibit broadened excitation profiles and high singlet oxygen production, enhancing the photodynamic inhibition efficacy against deep-tissue cancers 37.

#### 2.2.1 Improving Solubility & Tumor Accumulation

Recent advances highlight the potential of hybrid nanoparticles (HNPs) to address the longstanding challenges of porphyrin-based PDT, particularly their hydrophobicity, aggregation, and poor tumor selectivity. Letícia B. Silva *et al.* demonstrated the development of polymer-inorganic hybrid nanoparticles capable of efficiently encapsulating porphyrins and delivering them into cancer cells. These HNPs not only improved the solubility and stability of the photosensitizer but also enabled higher cellular uptake compared to free porphyrins. Upon light activation, the system generated significantly enhanced levels of ROS, resulting in superior cytotoxicity against tumor cells 38. This work illustrates how hybrid nanostructures can synergize the advantages of both organic and inorganic carriers, biocompatibility, stability, and multifunctionality to boost PDT outcomes. Such strategies lay the groundwork for the rational design of next-generation nanoplatforms that integrate imaging, targeting, and therapy in a single construct. These systems address solubility/aggregation issues, but the complexity of hybrid organic-inorganic constructs may slow regulatory approval compared to simpler liposomal formulations. This addresses the long-standing issue of porphyrin aggregation and poor solubility. Hybrid nanoparticles provide a stabilized, monodisperse system that enhances cellular uptake and ROS output. Yet, high formulation complexity may slow regulatory approval.

One of the significant limitations of porphyrin-based photosensitizers in PDT is their tendency to aggregate and their poor solubility in biological media. Silva *et al.* addressed this challenge by developing lipid-chitosan hybrid nanoparticles (P-HNPs) conjugated with porphyrins (∼130 nm). These P-HNPs significantly improved the solubility and stability of the photosensitizer while preventing self-aggregation. In bladder cancer cell models, the hybrid system enabled superior intracellular uptake and enhanced ROS generation under light irradiation, resulting in a 3.2-fold lower IC₅₀ compared with free porphyrin 39. This work demonstrates how hybrid nanostructures can synergistically integrate the biocompatibility of lipids with the stability of chitosan to achieve potent PDT activity. By preventing porphyrin self-aggregation, this system solves the reduced phototoxicity seen in earlier porphyrin formulations. It also improves intracellular retention. However, tumor-specific targeting is still limited, requiring further functionalization.

**Figure 8:**
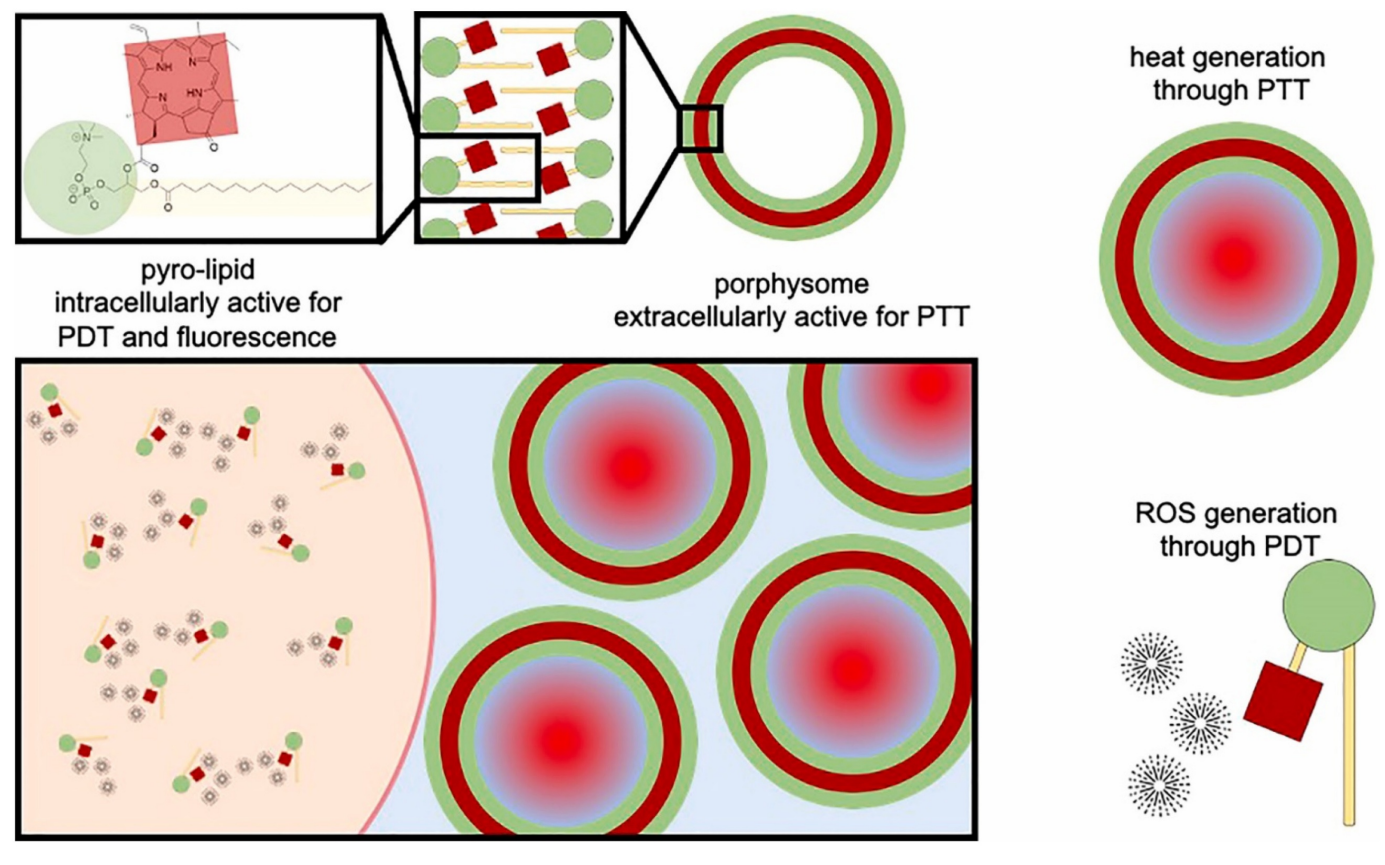
Porphysomes—self-assembled porphyrin-lipid nanovesicles as multifunctional nanocarriers. They improve photosensitizer solubility, stability, and tumor accumulation while enabling fluorescence/photoacoustic imaging and potent PDT efficacy *in vivo* 40. The figure was adapted from *Guidolin, K., et al., 2021* with permission from Wiley.

Liposomal carriers have long been studied as versatile delivery systems for hydrophobic drugs and photosensitizers. Guidolin *et al.* reported porphysomes, self-assembled vesicles composed entirely of porphyrin-lipid conjugates. In an A549 human lung tumor xenograft model, porphysome-based PDT (10 mg/kg, 671 nm irradiation) achieved marked tumor suppression and a 25% complete response rate, outperforming the clinical benchmark Photofrin® (15%) (Figure 8) 40. Beyond phototoxic efficacy, porphysomes also function as multimodal theranostic agents, combining fluorescence imaging, photoacoustic imaging, and therapeutic activity in a single nanoplatform. These findings highlight the potential of liposomal assemblies not only as drug carriers but also as self-contained photosensitizers with intrinsic imaging capabilities. Porphysomes address three limitations at once: photosensitizer instability, poor tumor accumulation, and lack of imaging capability. Their intrinsic photoacoustic and fluorescence properties allow simultaneous diagnosis and therapy. Challenges include immune clearance and long-term biodegradability. Clinically attractive due to intrinsic imaging + therapy, but stability and immune recognition remain concerns for systemic delivery. Comparative head-to-head studies vs clinical Photofrin® are still limited.

**Figure 9:**
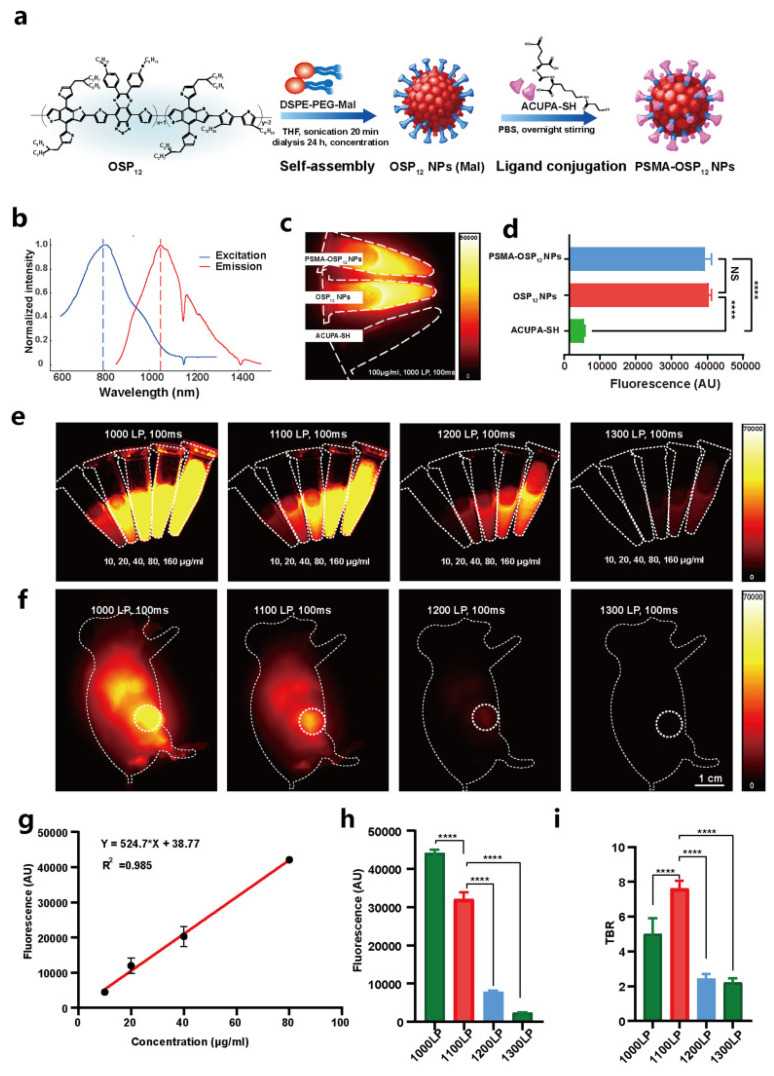
Rose Bengal-loaded chitosan nanoparticles for prostate cancer PDT. The organic nanocarrier improves solubility, stability, and tumor uptake of photosensitizers, significantly enhancing ROS-driven apoptosis compared with free RB 41. The figure was adapted from *Jiang, Z., et al., 2025* with permission from Dove Medical Press.

Encapsulating xanthene-type PSs into chitosan leverages both cationic charge and hydrogen bonding to stabilize payloads and promote uptake by prostate cancer cells. Uddin *et al.* formulated Rose Bengal-loaded chitosan NPs and showed significantly heightened phototoxicity against PC-3 cells versus free RB, with clear ROS-driven apoptosis under light, underscoring how even simple, fully organic matrices can convert a labile, self-quenching PS into a potent prostate-PDT agent (Figure 9) 41.

**Figure 10:**
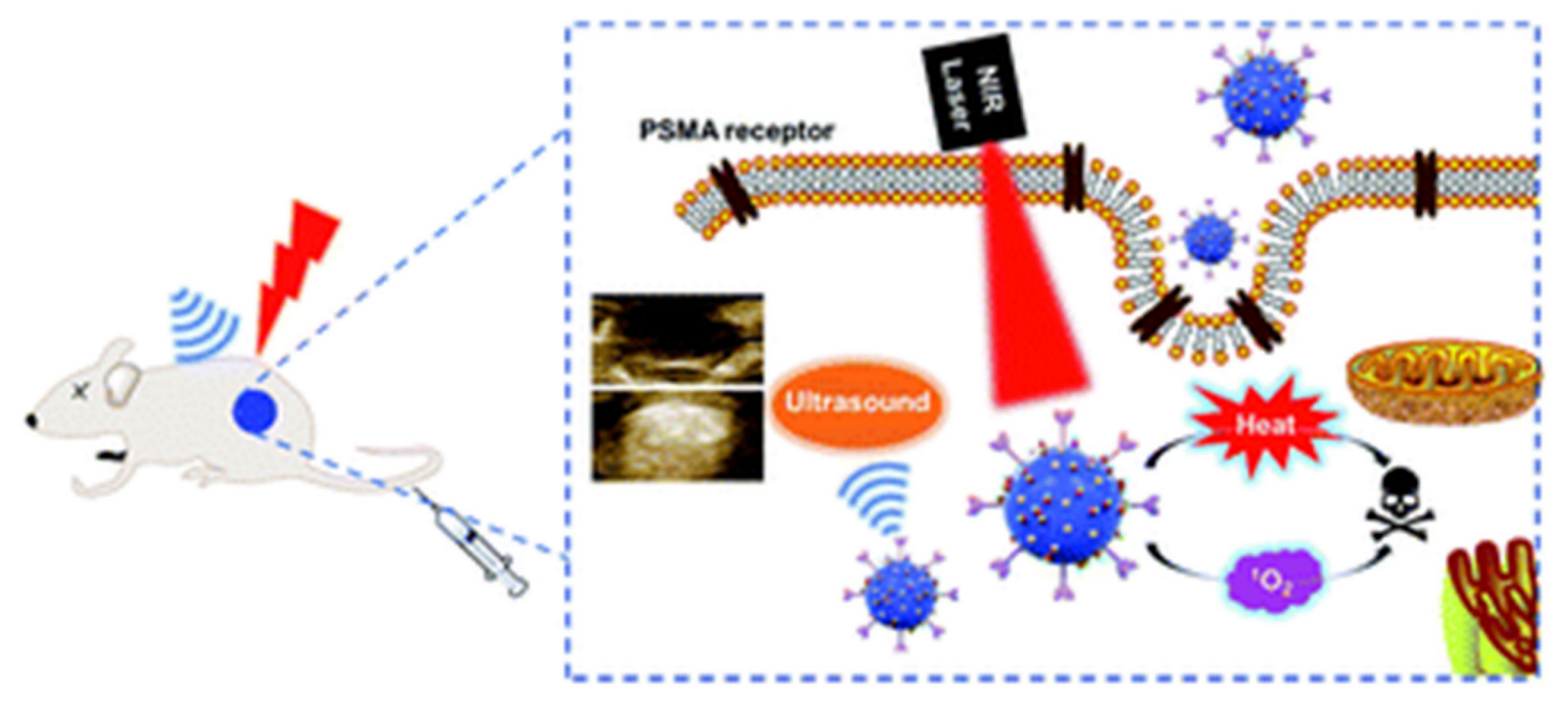
PDA-based multifunctional nanoparticles. Polydopamine enhances Ce6 loading, provides photothermal synergy, and enables multimodal imaging, yielding superior PDT-PTT efficacy in prostate cancer models 43. The figure was adapted from *Dai, L., et al., 2021* with permission from Royal Society of Chemistry.

Porphyrinic photosensitizers tethered to poly(vinylpyrrolidone) (PVP) covalent linking elevates aqueous stability, reduces aggregation, and improves cellular phototoxicity. Mesquita *et al.* reported PVP-porphyrin systems with enhanced PDT efficacy in prostate-cancer models, positioning PS-polymer conjugation as a robust route when bathochromic shift or monomeric PS behavior is needed under physiological conditions 42. Polydopamine (PDA) is a broadly biocompatible, π-rich polymer that adsorbs Ce6 noncovalently yet firmly, enabling high payload without chemical modification. It also contributes to photothermal conversion important for synergy (Figure 10) 43.

#### 2.2.2 Stimuli-Responsive / Pathway-Targeting Nanocarriers

**Figure 11:**
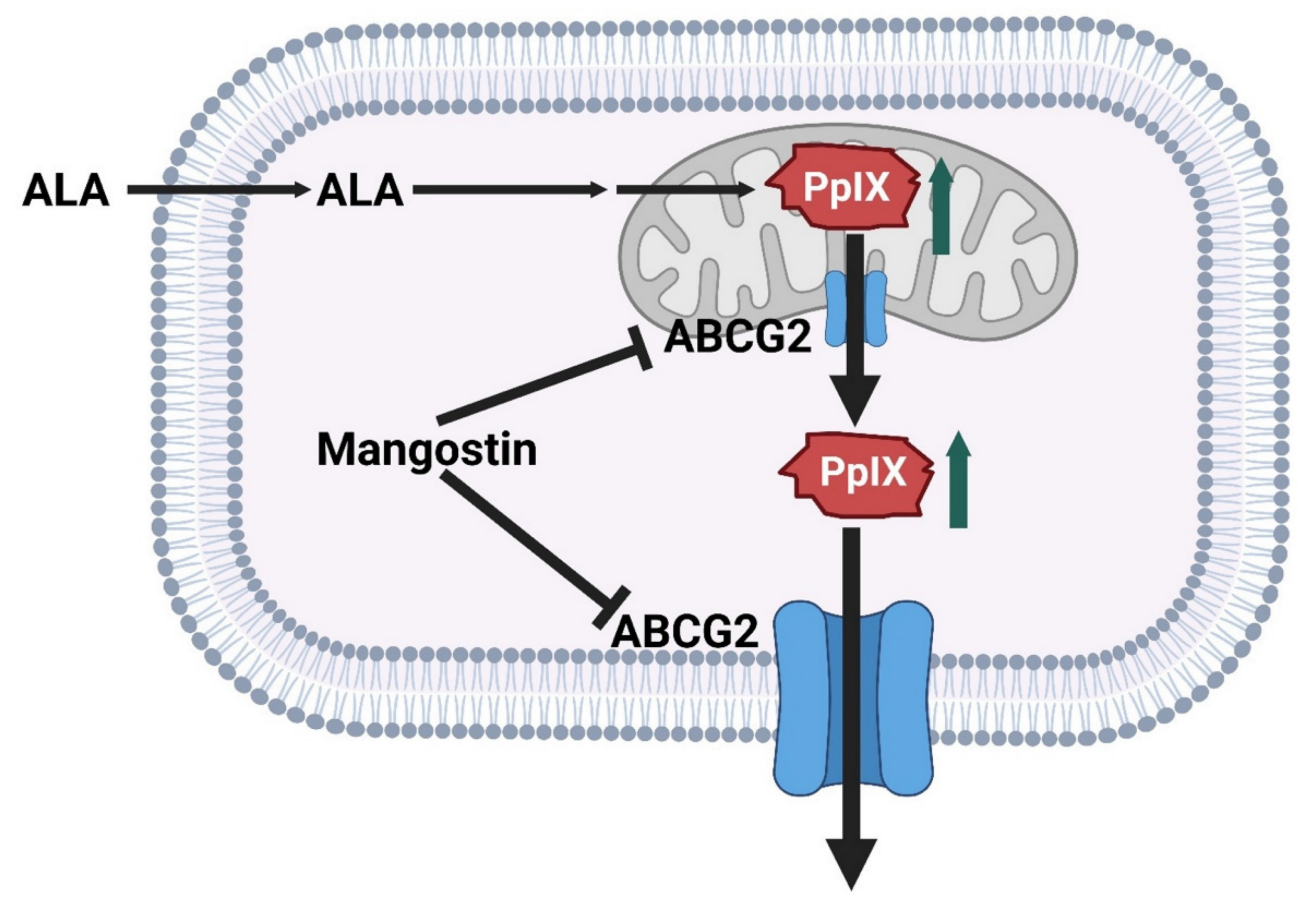
Mangostin as an ABCG2 inhibitor enhancing ALA-PDT. By blocking protoporphyrin IX efflux, mangostin increases intracellular photosensitizer retention, thereby amplifying ROS generation, apoptosis, and overall PDT cytotoxicity.

Recent studies have shown that mangostin, a bioactive xanthone derived from Garcinia mangostana, significantly enhances the therapeutic efficacy of aminolevulinic acid-based photodynamic therapy (ALA-PDT) in cancer treatment. ALA-PDT relies on the intracellular accumulation of PpIX, a photosensitizer generated through the heme biosynthetic pathway. However, the efficacy of this treatment is often compromised by the activity of ATP-binding cassette transporter G2 (ABCG2), which actively effluxes PpIX out of cancer cells, reducing intracellular retention and subsequent cytotoxic ROS generation upon light activation. Mangostin functions as a potent inhibitor of ABCG2, thereby preventing PpIX efflux and promoting its accumulation within tumor cells. This results in enhanced ROS production, greater induction of apoptosis, and improved overall anti-cancer activity when combined with ALA-PDT (Figure 11) 44. This study solves a clinically known resistance mechanism, PpIX efflux by inhibiting ABCG2. This increases intracellular PpIX levels and enhances PDT potency. The limitation is that mangostin has multiple off-target effects requiring careful dose optimization. Notably, this combinatorial approach selectively enhances cytotoxicity in malignant cells while sparing normal cells, underscoring its potential as a strategy to overcome resistance mechanisms and expand the clinical utility of PDT. Pharmacological inhibition of efflux transporters is a clever way to enhance PpIX, but mangostin's pleiotropic effects and pharmacokinetics require further exploration to ensure tumor selectivity.

**Figure 12:**
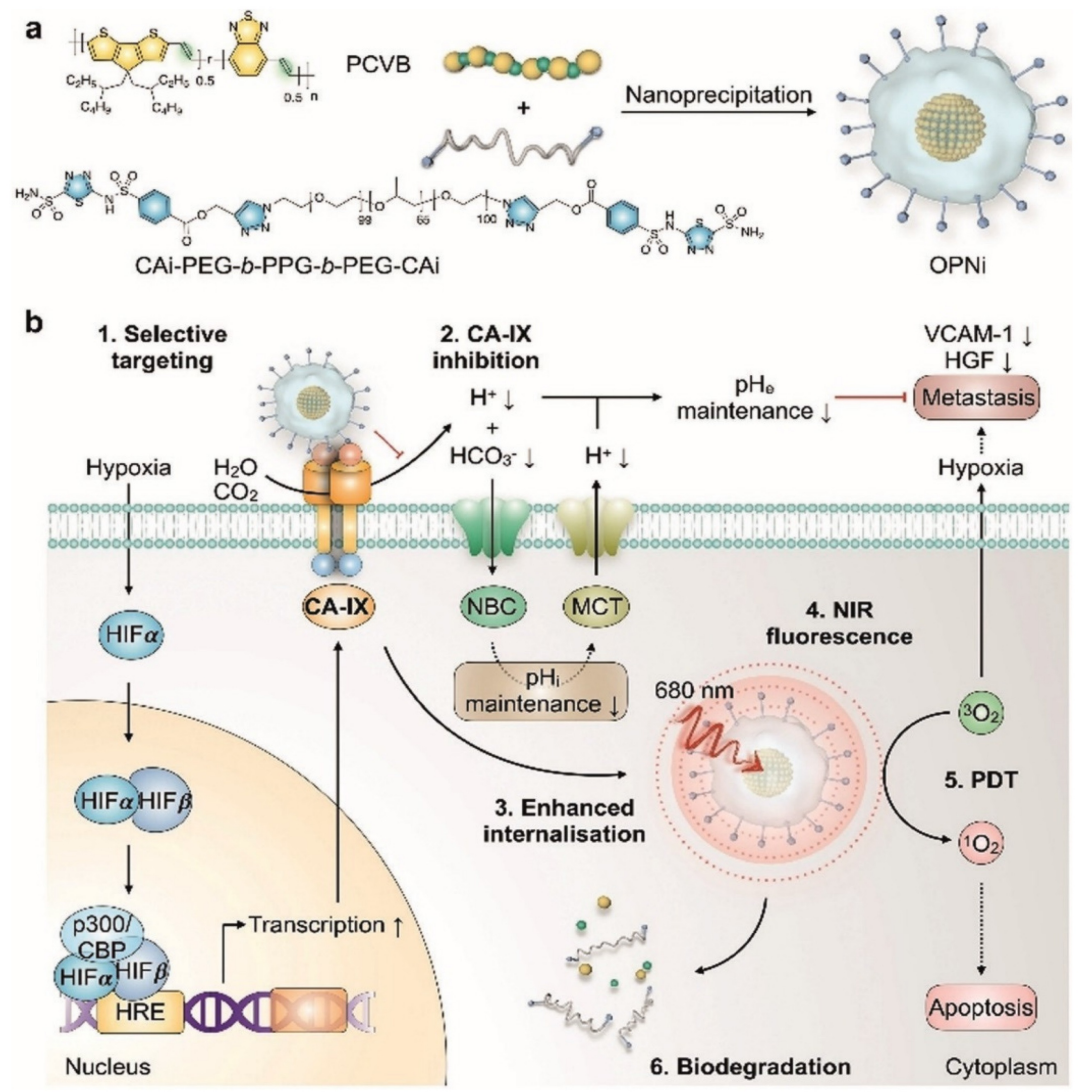
Organic photodynamic nanoinhibitors with dual action. These nanostructures generate ROS under light while simultaneously inhibiting tumor survival pathways, effectively overcoming resistance and enhancing PDT efficacy 45. The figure was adapted from *Jiang, Y., et al., 2019* with permission from John Wiley and Sons.

An emerging strategy in PDT research involves the development of organic photodynamic nanoinhibitors, which integrate photosensitization with complementary therapeutic modalities to achieve synergistic anticancer effects. Jiang *et al.* designed organic nanoinhibitors with intrinsic photodynamic activity that, upon light irradiation, not only generate ROS but also concurrently suppress tumor survival pathways. This dual functionality addresses one of the central limitations of conventional PDT, the rapid activation of tumor resistance mechanisms and hypoxia-driven escape routes. By engineering photosensitizers into organic nanoscale constructs, researchers achieved enhanced tumor accumulation via the EPR effect, improved photostability, and selective cellular uptake. More importantly, the nanoinhibitor framework enabled co-delivery of pathway-targeting agents that act in concert with PDT, leading to amplified oxidative stress and disruption of pro-survival signaling (Figure 12). In preclinical tumor models, this synergistic approach produced superior therapeutic efficacy compared to standalone PDT, highlighting the translational promise of organic photodynamic nanoinhibitors in overcoming tumor resilience 45. These nanoinhibitors solve the problem of rapid tumor adaptation by integrating pathway inhibition with PDT-derived ROS, addressing resistance that conventional PDT alone cannot overcome. However, multi-component systems increase formulation instability. These dual-function designs overcome resistance but add formulation complexity. It remains uncertain how regulatory pathways will view nanosystems that act simultaneously as drug + inhibitor. This research exemplifies the shift from monotherapy-based PDT to multifunctional phototheranostics, where the photosensitizer itself doubles as a therapeutic inhibitor. Such a strategy opens new avenues for precision oncology, offering a rationally designed, all-organic nanoplatform that integrates light-triggered cytotoxicity with molecularly targeted tumor suppression.

**Figure 13:**
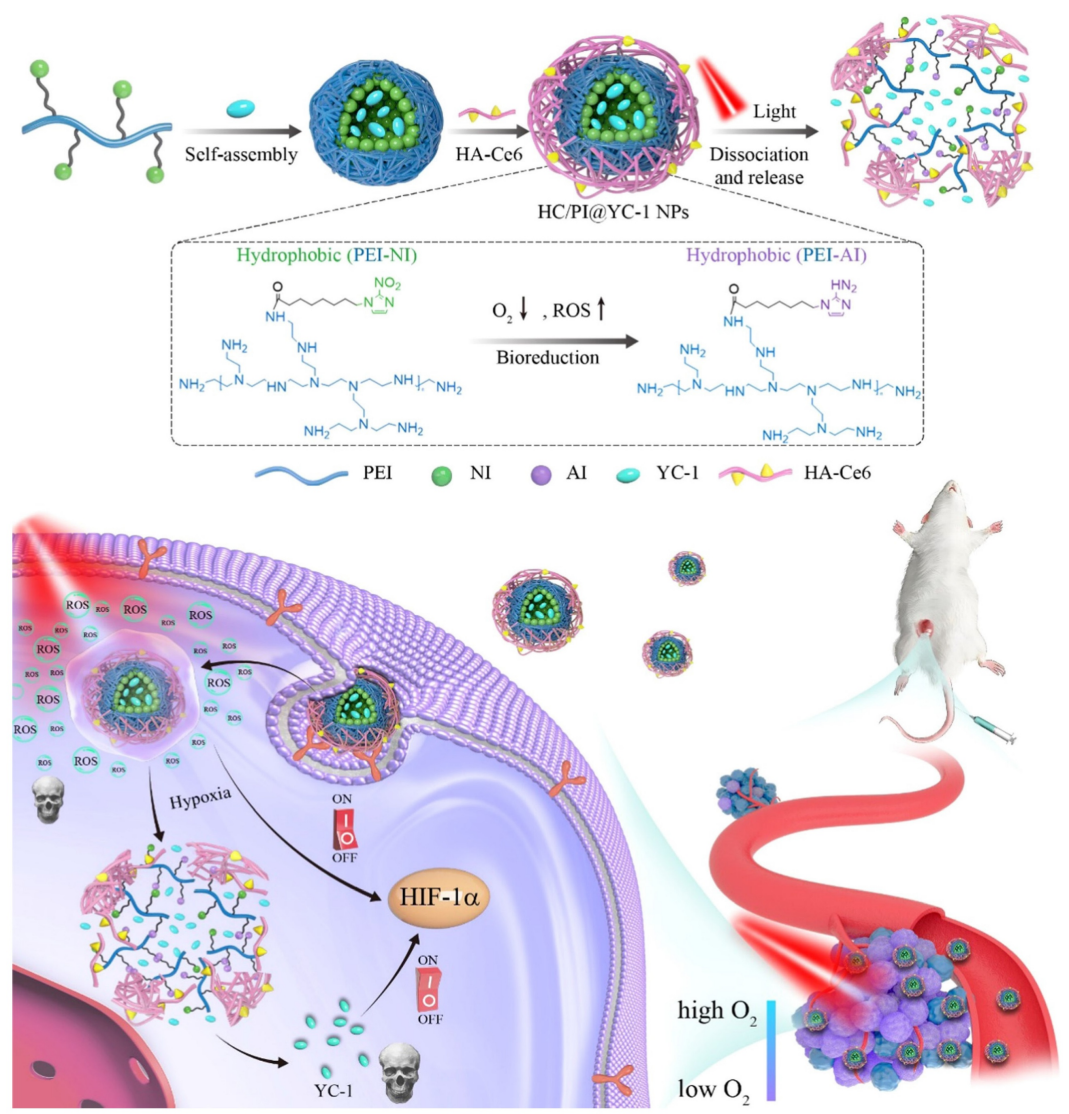
Hypoxia-adaptable chemo-photodynamic nanoinhibitors. The nanoplatform activates PDT under normoxic conditions, while hypoxia triggers the release of the alkylating agent HN2, allowing a switch from photodynamic action to hypoxia-activated chemotherapy and preserving cytotoxicity across heterogeneous oxygen environments. 46. The figure was adapted from *Wang, Y., et al., 2022* with permission from American Chemical Society.

A key challenge in conventional PDT lies in the oxygen dependence of ROS generation, which severely compromises therapeutic efficacy within hypoxic TME. To address this limitation, Wang *et al.* introduced self-rectifiable, hypoxia-assisted chemo-photodynamic nanoinhibitors that integrate oxygen-supply strategies with complementary chemotherapeutic functions (Figure 13). These smart nanoplatforms are designed not only to perform efficient PDT under normoxic conditions but also to adaptively activate therapeutic modules under hypoxia adaptively, thereby maintaining treatment efficacy across heterogeneous tumor niches 46. This work provides a dynamic solution to heterogeneous tumor oxygen levels. When oxygen is available, PDT dominates; under hypoxia, the chemical prodrug is activated. This overcomes the single-mode limitation of classical PDT. The drawback is the complexity of dual-responsive designs. *In vivo* investigations have demonstrated robust tumor suppression with reduced systemic toxicity, validating the translational potential of such nanoinhibitors. Beyond enhancing ROS-mediated killing, this strategy exemplifies how rational nanomedicine engineering can reprogram the TME to sustain therapy, setting a strong precedent for multi-functional, next-generation phototheranostic platforms. The “self-rectifiable” property arises from the dual-mode architecture of these nanoinhibitors. While the photodynamic pathway predominates under sufficient oxygen, a hypoxia-activated prodrug or molecular inhibitor is simultaneously incorporated to take over cytotoxic function when oxygen levels decline. This adaptive therapeutic switching ensures a continuous and potent anticancer effect, effectively overcoming the hypoxia-induced resistance that often limits the effectiveness of PDT. Furthermore, the nanoscale design enhances tumor accumulation and enables co-localized delivery of photodynamic and chemotherapeutic agents, resulting in pronounced synergism. Adaptive switching is conceptually elegant, but multi-component constructs face challenges in terms of reproducibility and stability in systemic circulation.

### 2.3. Overcoming Light Penetration and Hypoxia

#### 2.3.1 Upconversion & Alternative Light-Activation Technologies

Over the last decade, PDT has undergone a remarkable transformation from a niche phototoxic modality into a multifunctional and immune-potentiating cancer therapy. Advances in photosensitizers, nanoplatform engineering, and light-delivery strategies have undoubtedly expanded the reach of PDT; however, their translation into clinical oncology remains cautious. Several key bottlenecks persist. First, the limited and heterogeneous penetration of light in human tissues continues to confine PDT primarily to superficial or endoscopically accessible tumors, despite creative solutions such as UCNPs and X-ray-activated scintillators. Recent advances have highlighted MnO₂ nanoflowers as an emerging multifunctional nanoplatform for cancer theranostics. Their unique flower-like morphology provides a large surface area and efficient reactivity within the TME. MnO₂ can decompose endogenous H₂O₂ to generate oxygen, thereby alleviating tumor hypoxia and significantly enhancing the efficacy of PDT. Simultaneously, their strong NIR absorption enables efficient photothermal conversion for PTT. Notably, the release of Mn²⁺ ions also offers T1-weighted MR imaging capability, allowing real-time tumor diagnosis and treatment monitoring. This integrated platform thus combines oxygen modulation, dual-mode therapy (PDT + PTT), and diagnostic imaging into a single system, making MnO₂ nanoflowers a promising candidate for next-generation cancer theranostics 23. Multifunctionality is impressive, but the release of Mn² raises long-term safety concerns. Second, hypoxia a universal feature of solid tumors remains a primary barrier that restricts ROS-dependent cytotoxicity. Although MnO₂ nanoplatforms, oxygen nanocarriers, and hypoxia-bypassing chemistries are promising, their long-term biosafety and reproducibility have not been systematically addressed. Third, the majority of nanocarrier systems that report outstanding preclinical efficacy often fail to demonstrate scalable manufacturing or regulatory readiness, underscoring the gap between innovation and translational pragmatism.

#### 2.3.2 Hypoxia-Relief & Oxygen-Supplying Nanoplatforms

Hypoxia is a first-order limiter for PDT in prostate tumors. Among OPNP solutions, PFPE-based Ce6 nanoemulsions stand out for their simplicity and translational logic dissolving O2 directly in a biocompatible fluoropolymer core, improving ROS yield *in vitro* (including PC-3) and *in vivo*. Pairing O2-rich cores with Prostate-Specific Membrane Antigen (PSMA) targeting or with PDA-PTT heating could further offset oxygen debt by boosting blood flow and ROS chemistry. Compared with traditional perfluorocarbon emulsions, PFPE cores exhibit approximately 2-3-fold higher oxygen solubility due to the increased fluorine content and lower viscosity of the polymeric matrix, which enhances O₂ dissolution and retention under physiological conditions. This superior O₂-loading efficiency underpins their advantage in sustaining ROS generation during PDT, even within severely hypoxic prostate tumor niches 47. Beyond first-generation NPs, NIR-II prostate probes and polymeric theranostics are emerging to push imaging depth and surgical guidance; when married to OPNP-PDT, this could sharpen lesion localization and margin control in focal therapy 41.

**Figure 14:**
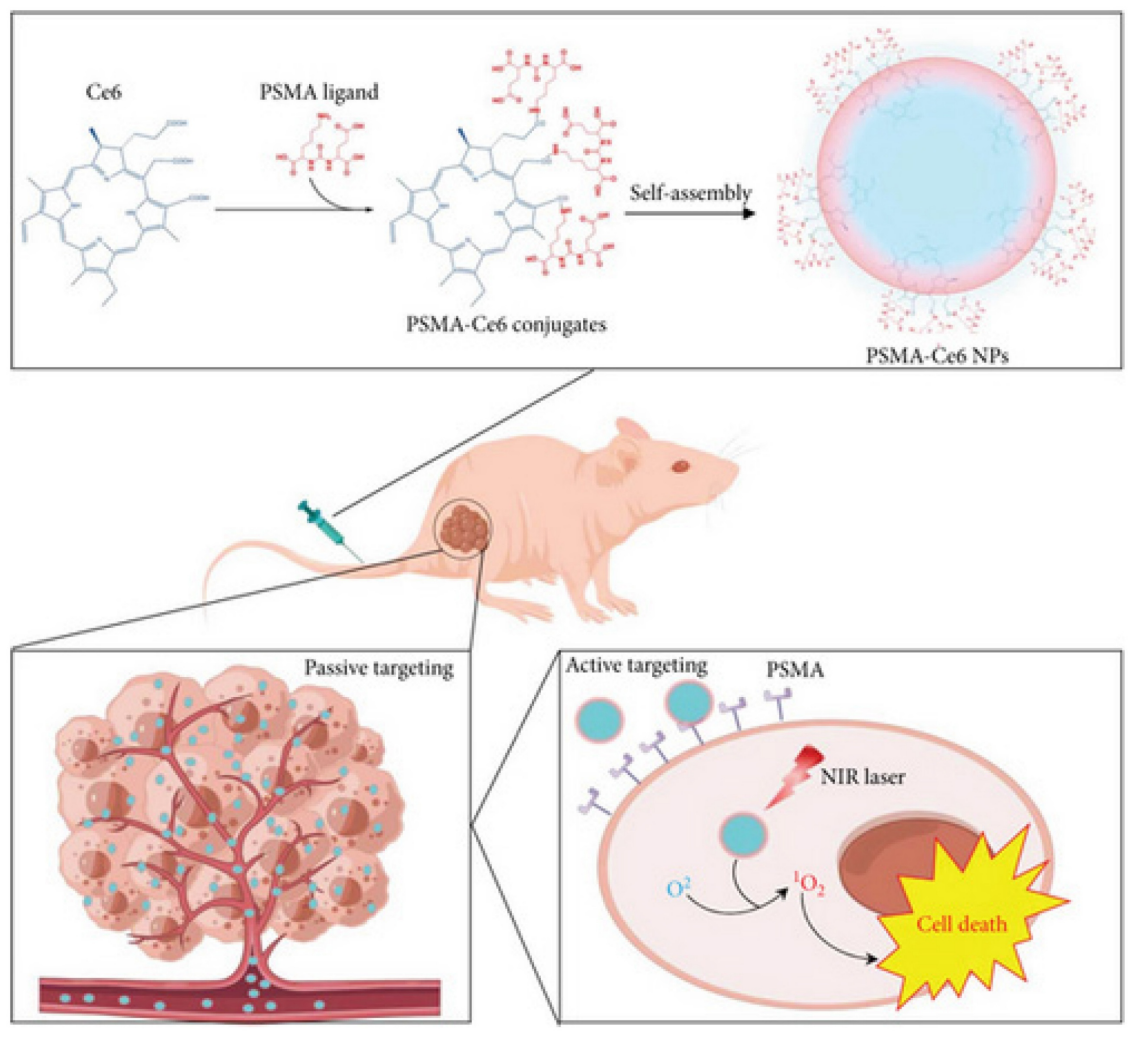
PSMA-targeted Ce6 nanoparticles for prostate cancer PDT. Ligand-mediated uptake improved tumor selectivity and phototoxicity in PSMA-positive tumors, demonstrating targeted delivery as a route to precision PDT 48. The figure was adapted from Deng*, Y., et al., 2022* with permission from John Wiley and Sons.

Deng *et al.* constructed PSMA-targeted Ce6 NPs that achieved preferential uptake and stronger PDT killing in PSMA-positive LNCaP over PSMA-negative controls, directly linking ligand density to prostate selectivity (Figure 14) 48. This study overcomes nonspecific uptake by exploiting PSMA expression in prostate tumors, greatly improving tumor selectivity. However, reliance on PSMA expression means limited applicability in PSMA-low cancers.

**Figure 15:**
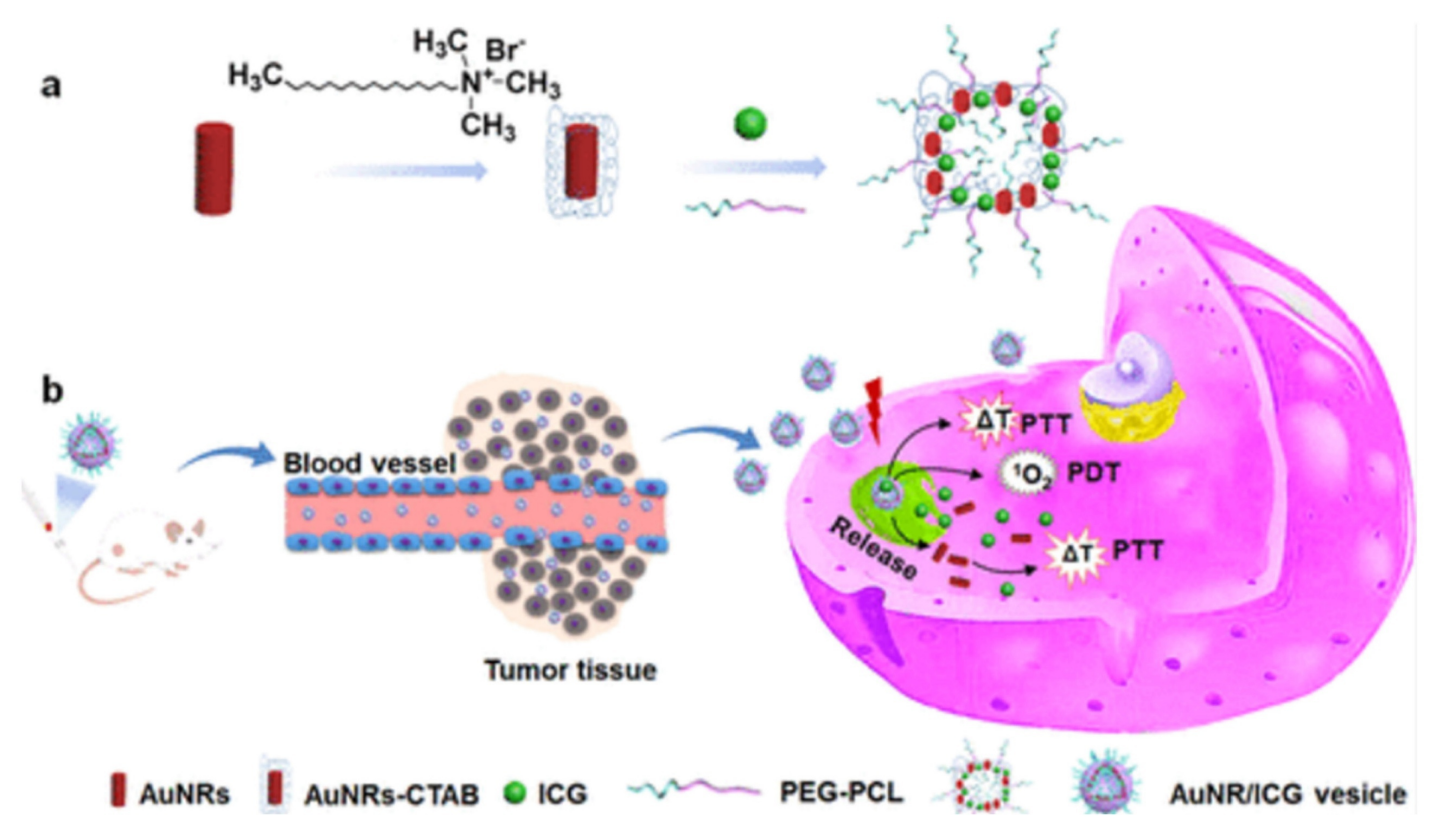
ROS-responsive polymeric vesicles (IIMS) for event-reporting PDT. ROS generated during PDT induces vesicle disassembly, resulting in payload release and activation of ICG fluorescence. This fluorescence increase provides real-time feedback on therapeutic activity and vesicle degradation 49. The figure was adapted from Hu*, J., et al., 2020* with permission from American Chemical Society.

ROS-responsive polymeric vesicles (ICG-bearing, membrane-mimetic vesicles) with organic shells can both deliver and *report* PDT activity, turning on fluorescence in oxidative niches and disassembling to release payloads. While demonstrated outside prostate models, Hu *et al.*'s IIMS vesicles highlight design rules for event-reporting OPNPs that could verify light dose/ROS in real time during prostate PDT (Figure 15) 49.

**Figure 16:**
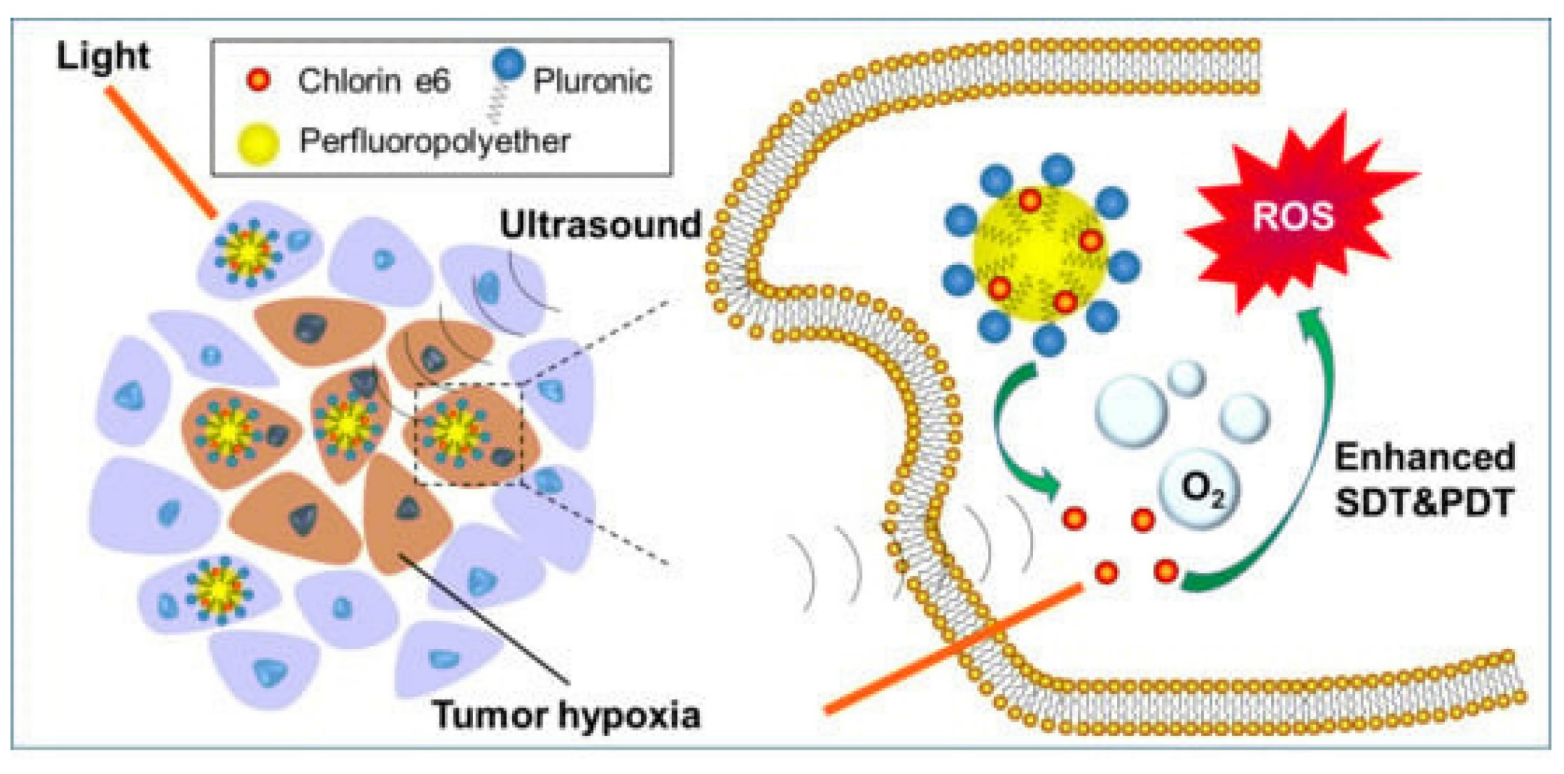
Oxygen-rich Ce6 nanoemulsions (PFPE-based) for hypoxia relief in PDT. The platform dissolves oxygen in a biocompatible core, sustaining singlet oxygen production in hypoxic tumors and improving therapeutic efficacy 47. The figure was adapted from Hong*, L., et al., 2020* with permission from MDPI.

Perfluoropolyether (PFPE)-based Ce6 nanoemulsions represent a pragmatic hypoxia-mitigating OPNP: they solubilize Ce6, carry dissolved O2, and preserve singlet-oxygen generation even in low oxygen. Hong *et al.* validated PC-3 cell phototoxicity and superior antitumor activity across models, spotlighting oxygen-replenishing polymers as a high-leverage fix for hypoxic prostate tumors (Figure 16). Compared with traditional perfluorocarbon emulsions, PFPE cores exhibit approximately 2-3-fold higher oxygen solubility due to the increased fluorine content and lower viscosity of the polymeric matrix, which enhances O₂ dissolution and retention under physiological conditions 47. This superior O₂-loading efficiency underpins their advantage in sustaining ROS generation during PDT, even within severely hypoxic prostate tumor niches.

**Figure 17:**
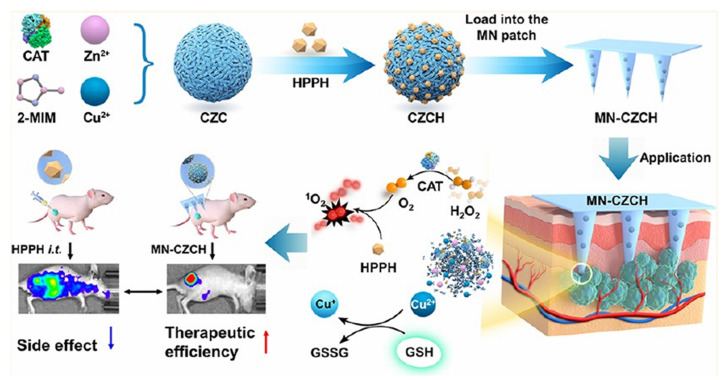
Microneedle (MN) patch integrating oxygen generation and glutathione depletion. The minimally invasive platform alleviates tumor hypoxia, weakens redox defenses, and enables repeatable PDT with enhanced tumor regression 17. The figure was adapted from *Li, Y., et al., 2022* with permission from American Chemical Society.

Y. Li *et al.* introduced an innovative microneedle (MN) patch-based platform designed to overcome two significant barriers in PDT: tumor hypoxia and intracellular antioxidant defense. The microneedle patch incorporates a self-oxygenating component that continuously generates oxygen at the tumor site, thereby alleviating the hypoxic microenvironment, which would otherwise limit PDT efficacy. In parallel, the system depletes intracellular glutathione (GSH), weakening the tumor's redox defense and enhancing ROS-mediated cytotoxicity. Highly patient-friendly and scalable, but repeated application in humans must confirm skin tolerability and local inflammation risks. *In vivo* experiments demonstrated that this dual-functional MN patch ensures deep, localized, and repeatable delivery of photosensitizers with minimal invasiveness. Compared with conventional PDT, treatment with the MN patch significantly enhanced ROS production, induced stronger tumor regression, and prolonged survival in animal models (Figure 17). Furthermore, the patch's ability to support repeatable treatment cycles without substantial side effects makes it a highly versatile strategy for long-term cancer management 17. This solves real-world clinical challenges: poor intratumoral penetration of PSs and hypoxia. MN patches deliver PS + oxygen directly into the tumor with minimal invasiveness. Limitations include skin irritation and difficulty treating deeply located tumors. This research highlights that this approach not only improves therapeutic precision but also represents a scalable and patient-friendly delivery system, bridging the gap between laboratory design and clinical translation of PDT.

Zhang *et al.* investigated the synergistic interaction between PDT and conventional chemotherapy in the treatment of skin cancers, with a particular focus on the role of autophagy regulation. PDT not only induces oxidative stress and apoptosis but also modulates autophagy pathways within tumor cells. When combined with chemotherapeutic drugs, PDT-mediated autophagy regulation sensitizes cancer cells, thereby overcoming resistance mechanisms that typically limit chemotherapy efficacy. *In vivo* and *in vitro* experiments demonstrated that the combination of PDT and chemotherapy leads to enhanced tumor growth inhibition, higher rates of cell death, and reduced recurrence compared to either treatment alone. Mechanistically, PDT has been shown to impair pro-survival autophagy and promote autophagic cell death, thereby amplifying the cytotoxic effects of chemotherapy agents 50. Overall, the study highlights that targeting autophagy is a promising strategy to potentiate chemotherapy outcomes in skin cancers, establishing PDT as both a direct cytotoxic modality and an adjuvant enhancer of therapeutic efficacy.

### 2.4. Combination Therapies

Combination therapies using PDT are promising for providing more effective cancer treatments. These therapies allow for overcoming the limitations of traditional treatments by offering a holistic approach that integrates various therapeutic mechanisms. The combination of PDT with chemotherapy offers a multi-pronged approach by employing both photodynamic processes and cytotoxic drugs. This combination can reduce recurrence rates and improve overall response in various cancer types like lung, breast, and cervical cancers. By integrating these two modalities, the therapeutic impact is enhanced, allowing for the overcoming of drug resistance and increasing target specificity.

#### 2.4.1 PDT + Immunotherapy

**Figure 18:**
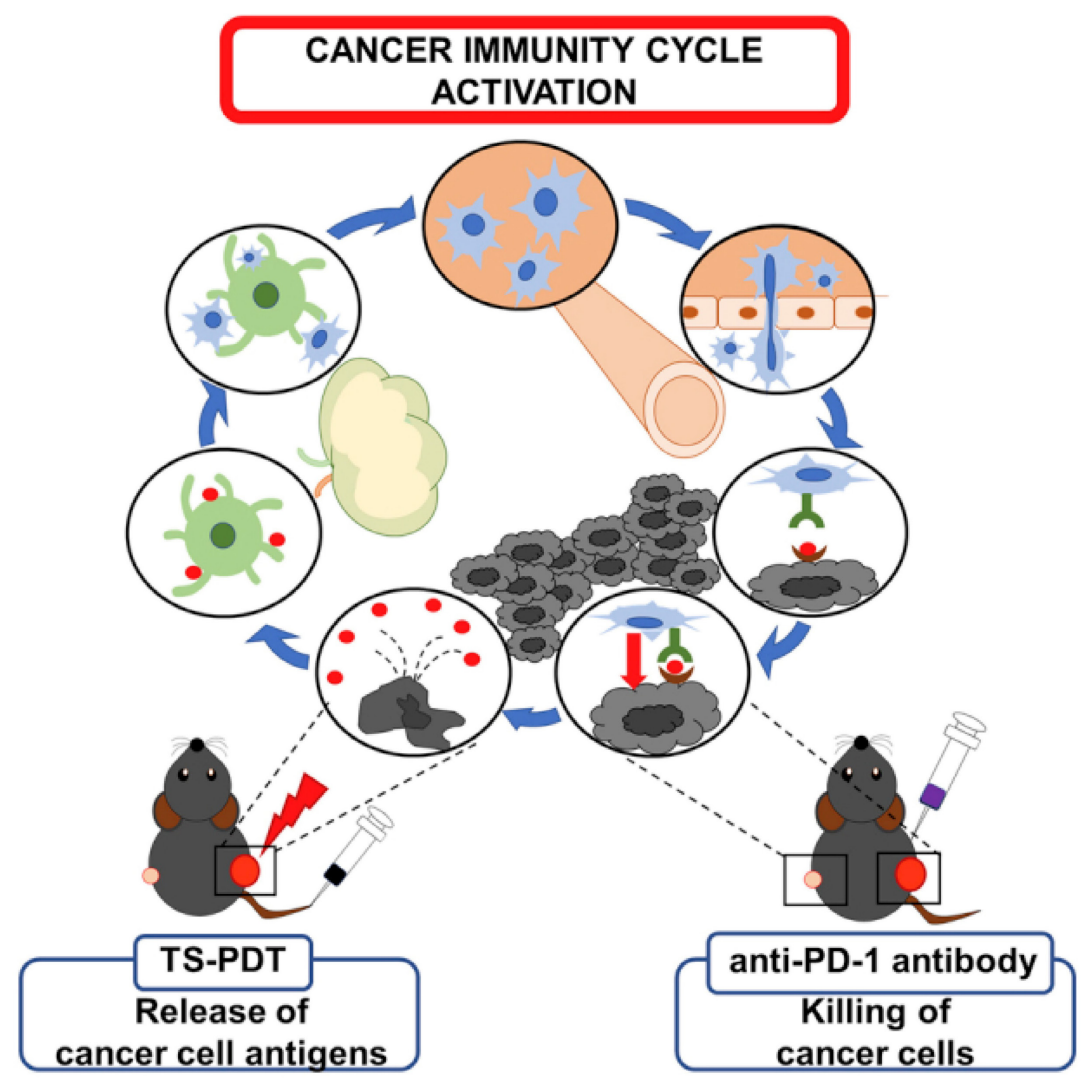
Checkpoint blockade combined with PDT. PDT-induced ICD enhances antigen release and immune priming, while immune checkpoint inhibitors strengthen T-cell activation, producing durable systemic tumor immunity. The figure was adapted from Li*, J., et al., 2025* with permission from John Wiley and Sons.

PDT can also facilitate the enhancement of immune system responses by promoting the release of tumor antigens and danger signals. When combined with ICB, PDT can enhance T-cell function, offering a powerful synergy in treating tumors that do not respond well to single therapies alone. This combination can lead to a more robust and sustained immune response against cancer cells. Progress in the field has further driven the exploration of immuno-photodynamic therapy (IPDT), where photosensitizers activate immune responses while minimizing residual photosensitizer toxicity after treatment. The use of H2O2-responsive aggregation-induced emission photosensitizers, such as TBZPYBE, improves therapeutic outcomes and safety after surgery in cancer treatments by promoting dendritic cell maturation and polarizing macrophages (Figure 18) 51. Demonstrates strong synergy, but dosing schedules and sequencing need optimization. Potential additive toxicities must be carefully balanced in clinical trials. An oxygen-economized PDT platform (NIR-excited) preserved ROS generation under hypoxia and elicited ICD *in vitro* and *in vivo*, offering a route to overcome the hypoxic barrier that often blunts both PDT cytotoxicity and innate immune activation in solid tumours.

Combining PTT with PDT addresses the limitation of hypoxia by enhancing the therapeutic efficacy through simultaneous heat generation and ROS production. PTT can significantly elevate the local temperature within tumors, promoting a synergistic effect when used with PDT. This combination has shown promise in achieving substantial tumor reduction. Another innovative approach involves combining PDT with other treatments, such as PTT. Smart platform designs, such as BOD-D, enable a switch from NIR-I imaging-guided PDT to NIR-II-guided PTT while releasing nitric oxide for gas therapy. This combined approach effectively addresses the limitations posed by hypoxic and complex TME 52.

Interstitial photodynamic therapy (I-PDT) has emerged as a promising minimally invasive modality for treating locally advanced cancers that are not amenable to surgical resection or conventional treatments and emphasizes the development and application of *in vivo* tumor models to investigate the therapeutic efficacy, dosimetry, and biological responses of I-PDT. Preclinical models, including orthotopic and xenograft tumor systems, offer a realistic platform for studying light distribution, photosensitizer uptake, and oxygen consumption within the TME. These models are crucial for optimizing treatment parameters such as light fluence, fiber placement, and drug-light intervals. Results demonstrated that I-PDT induces significant tumor necrosis and vascular shutdown, with therapeutic outcomes strongly dependent on precise dosimetric control and facilitate the prediction of treatment response and recurrence patterns, thereby bridging the gap between experimental studies and clinical applications. Notably, the work underscores that animal models provide valuable insights into tissue optical properties and heterogeneity of tumor vasculature, which are critical determinants of I-PDT success 53. The study concludes that robust *in vivo* cancer models are indispensable for translating I-PDT into clinical practice, enabling refinement of treatment strategies and paving the way for its integration into multimodal cancer therapies.

**Figure 19:**
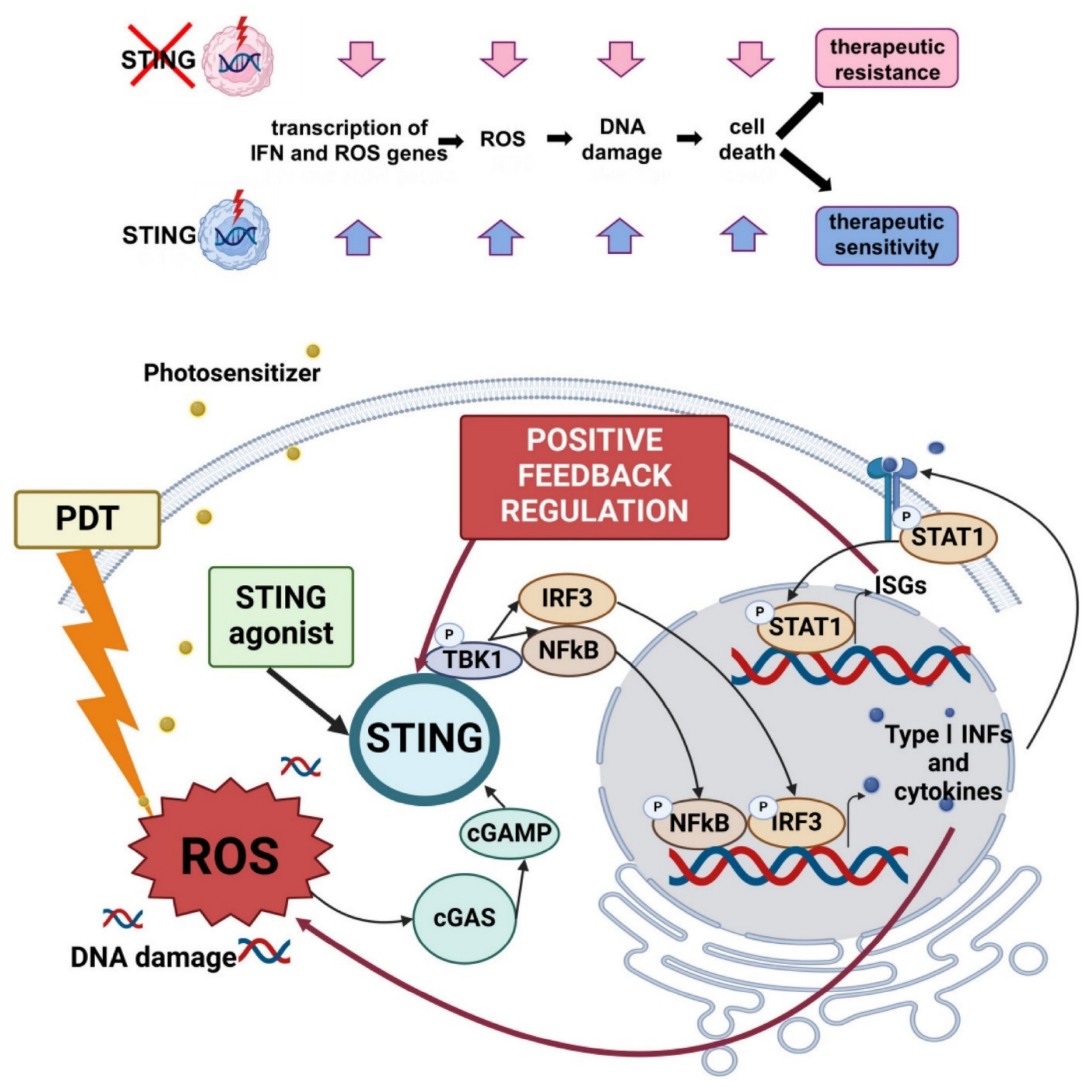
cGAS-STING pathway activation by PDT. Talaporfin sodium PDT enhances innate sensing and type I interferon signaling, amplifying antitumor immune responses and improving synergy with immunotherapy 64. The figure was adapted from Sasaki*, M., et al., 2025* with permission from Japanese Cancer Association; Blackwell Publishing.

PDT has re-emerged as a precision immuno-oncology modality, beyond local cytotoxicity. Properly dosed PDT converts “cold” tumors into “hot,” T-cell-infiltrated lesions by inducing ICD, exposing tumor antigens, and revamping antigen-presenting cell (APC) function. Over the last five years, three translational arcs have dominated: (i) engineering PDT to deepen ICD and innate sensing (cGAS-STING), (ii) integrating PDT (and antibody-targeted photoimmunotherapy) with ICB to secure abscopal control and memory, and (iii) building *in situ* cancer vaccines by co-delivering photoactivatable adjuvants. Multiple recent studies demonstrate that modern photosensitizers and dosing regimens intensify hallmark ICD signals (ecto-calreticulin, HMGB1/ATP release) and couple them to cytosolic DNA sensing via cGAS-STING, amplifying type I interferon and cross-priming. In mouse models, talaporfin-based PDT (TS-PDT) was shown to enhance STING-dependent signalling; genetic STING loss abrogated PDT-driven tumour control, while adding a pharmacologic STING agonist further improved outcomes versus either monotherapy, supporting a direct mechanistic link between ROS, STING tone, and antitumour immunity (Figure 19) 54.

Across syngeneic models (melanoma, pancreatic), chlorin e6 (Ce6)-PDT enhances PD-L1/PD-1 pathway dependence and improves responses to anti-PD-1 therapy, with increased CD8⁺ T-cell infiltration and abscopal suppression of untreated lesions. Ce6-PDT reprograms both irradiated and distant tumours, functionally “priming” ICB 55. NIR photoimmunotherapy (NIR-PIT) antibody-photoabsorber conjugates (PD-L1-targeted) have matured into a potent, antigen-specific variant of PDT that preferentially kills target-antigen⁺ cells and elicits robust immune activation. In immune-competent mice, PD-L1-targeted NIR-PIT produced strong local control, abscopal effects, and durable memory; feasibility data now support combining NIR-PIT with checkpoint inhibitors in unresectable recurrent head & neck cancer. Preclinical PDX work and bibliometric analyses further indicate rapid expansion of NIR-PIT's immune-oncology footprint across solid tumours 56.

Rakuten Medical presented preclinical data on a novel PD-L1-targeted photoimmunotherapy agent (RM-0256), an anti-PD-L1 antibody conjugated to the photoabsorber IR700. Unlike conventional PD-L1 blockade, RM-0256 selectively destroyed PD-L1-expressing tumor cells and immunosuppressive myeloid cells (tumor-associated macrophages and myeloid-derived suppressor cells) upon NIR irradiation. In mouse tumor models resistant to checkpoint inhibitors, RM-0256 significantly suppressed tumor growth, enhanced CD8⁺ T-cell infiltration, and remodeled the immunosuppressive TME. Significantly, the treatment outperformed anti-PD-L1 antibody therapy alone, offering a dual mode of action: immune checkpoint interference and photo-induced cytotoxicity. Early good laboratory practice (GLP) toxicity studies in cynomolgus monkeys suggested favorable safety and tolerability, strengthening the translational promise of PD-L1-directed photoimmunotherapy as a next-generation immuno-oncology approach 57.

**Figure 20:**
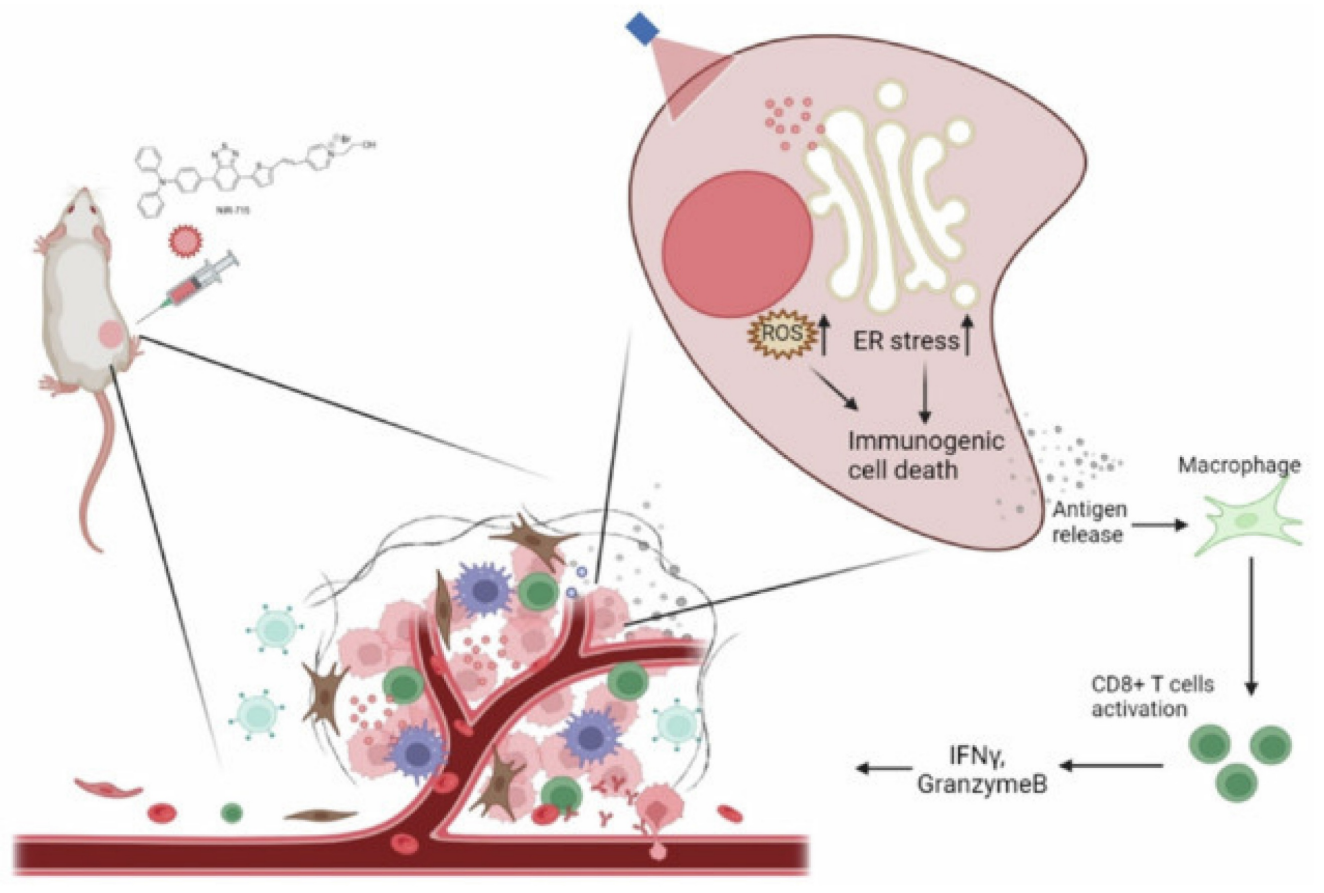
Schematic illustration of NIR-715 PDT for visible light-triggered, endoplasmic reticulum-targeting antitumor therapy 58. The figure was adapted from *Zheng, Z.Y., et al., 2024* with permission from Springer Nature.

Zheng *et al.* designed an organelle-specific PDT strategy using **NIR-715**, a photosensitizer targeted to the endoplasmic reticulum (ER). This localization selectively induced ER stress upon irradiation, thereby amplifying ICD and releasing strong “eat-me” signals that activated DCs. The treatment reshaped the tumor immune microenvironment by promoting CD8⁺ T-cell infiltration and enhancing responsiveness to checkpoint inhibitors. In murine tumor models, NIR-715 PDT not only eradicated primary tumors but also conferred durable systemic immunity against rechallenge (Figure 20). Compared to conventional photosensitizers, ER-targeted NIR-715 displayed superior immune activation with minimal off-target damage, making it a promising design principle for next-generation PDT platforms 58.

**Figure 21:**
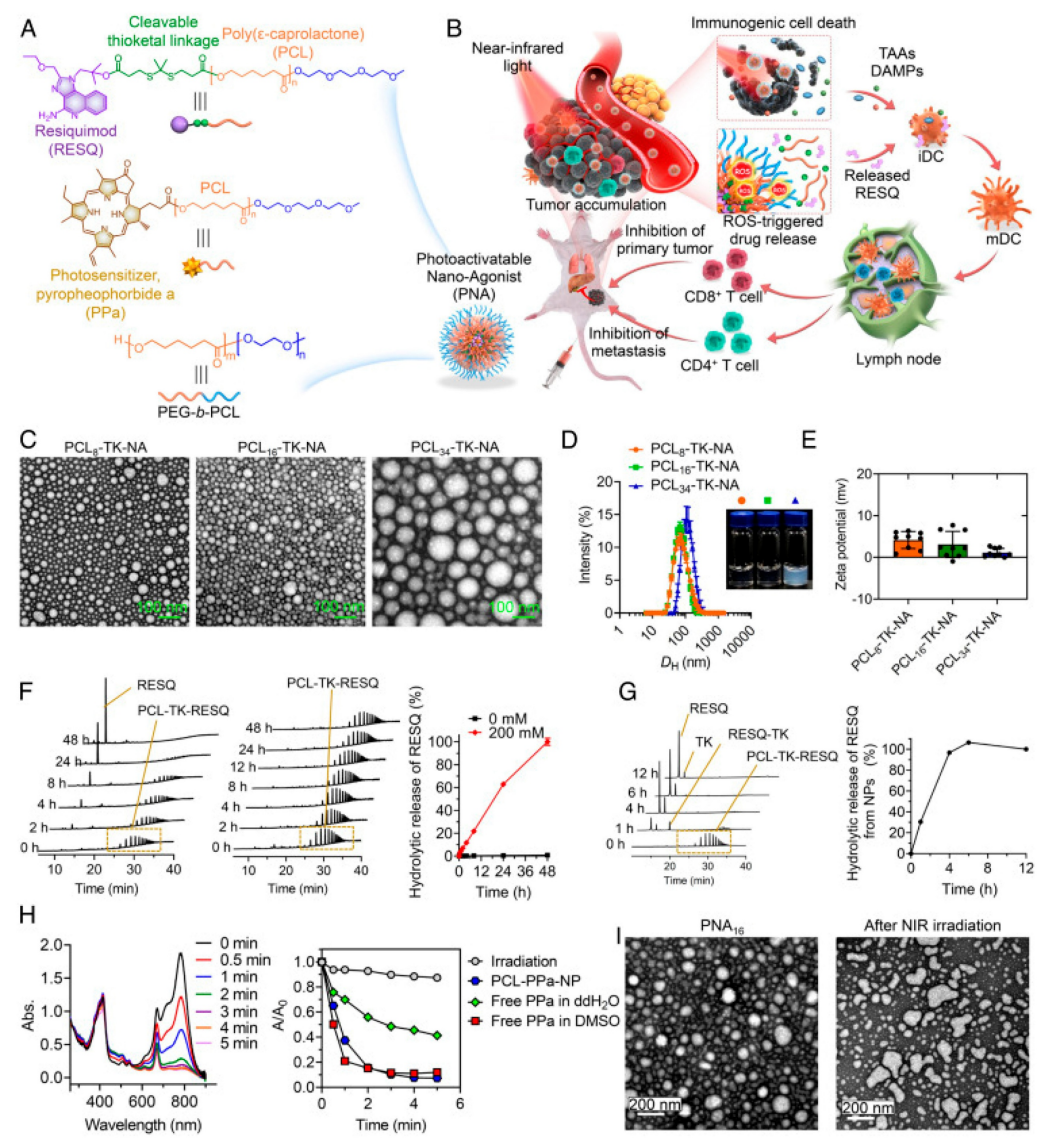
Photoactivatable nanoagonists (PNAs) combining PDT with immunotherapy. NIR-triggered ROS generation induces ICD, while ROS-cleavable linkers release TLR agonists, transforming PDT into an *in situ* cancer vaccine 59. The figure was adapted from Wan*, J., et al., 2023* with permission from National Academy of Sciences.

Wan *et al.* reported the development of *photoactivatable nanoagonists (PNAs)* as a programmable platform to combine photodynamic tumor ablation with immune adjuvant delivery. Their nanostructure was composed of a photosensitizer covalently linked to the Toll-Like Receptor (TLR), TLR7/8 agonist resiquimod (R848) via a ROS-cleavable linker, enabling controlled drug release upon NIR light activation. Once irradiated, PNAs produced ROS that triggered ICD in tumor cells, while simultaneously liberating R848 to activate DCs. This dual-mode action remodeled the TME, promoted robust CD8⁺ T-cell priming, and conferred durable antitumor memory responses in multiple murine cancer models. Importantly, PNAs demonstrated strong suppression of local tumor recurrence and distant metastasis, highlighting their potential as a modular *in situ* cancer vaccine (Figure 21) 59.

#### 2.4.2 PDT + Photothermal Therapy (PTT)

Dai *et al.* engineered Ce6@PDA-DCL-PFP OPNPs that pair PSMA targeting (DCL), perfluoropentane (PFP) for ultrasound contrast, and PDA's inherent photothermal effect. In comparison to the non-targeted control, the targeted OPNPs exhibited markedly higher cellular uptake and tumor accumulation, enabling precise US guidance and more complete ablation under dual-wavelength irradiation *in vivo*. Flow-cytometry and imaging further confirmed lower uptake in PSMA-negative PC-3, a helpful internal control for specificity 43. This is one of the most advanced niches, characterized by clear design rules. However, most data are still preclinical and heavily reliant on xenograft models. Orthotopic and PSMA-heterogeneous tumors should be prioritized for more clinically realistic validation. PSMA ligands on organic polymer backbones (PDA, amphiphiles, PEGylated blocks) consistently increase tumor selectivity and treatment indices for prostate PDT, and they can be combined with imaging handles (US microbubble precursors) 60. This platform simultaneously solves hypoxia (O₂ core), poor targeting (PSMA ligand), low ROS yield (Ce6), and shallow penetration (dual-wavelength PDT/PTT). Remaining limitations include formulation complexity and potential immune recognition.

**Figure 22:**
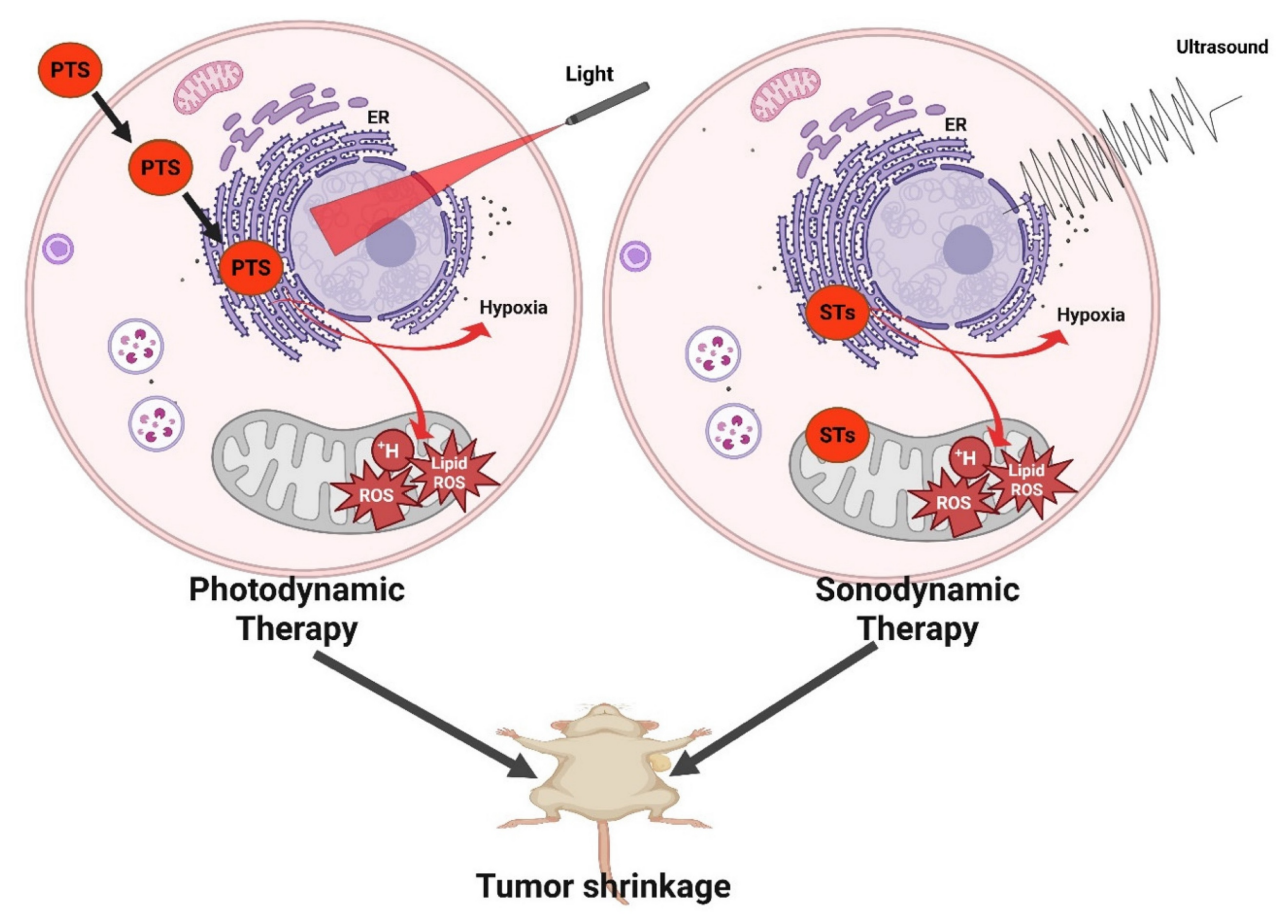
Ce6@PDA-DCL-PFP oxygenated nanoparticles integrating PDT, PTT, and ultrasound imaging. The multifunctional system combines hypoxia relief, dual-wavelength ablation, and image guidance, enabling complete tumor eradication *in vivo*.

Ce6@PDA-DCL-PFP uniquely combines Ce6-PDT (660 nm) with PDA-PTT (808 nm), enabling sequential or simultaneous light regimens for deeper, more uniform ablation and reduced recurrence, making it an especially compelling proposition for multifocal or hypoxic lesions. The same platform provides *in situ* ultrasound contrast, improving intra-procedural guidance. This tri-functional integration (targeting + therapy + imaging) is challenging to replicate with low-MW PS alone 43. Event-reporting OPNPs (ROS-responsive vesicles) further reinforce the “theranostic” loop, connecting delivered light, ROS generation, and payload release to the actual TME functionality that could be translated to the prostate for on-table verification of adequate PDT dose (Figure 22) 49. A smart drug-repurposing strategy, but a trade-off in selectivity (normal/cancer toxicity ratio drop) needs to be resolved before clinical prioritization.

Ce6@Prostate tumors are anatomically tucked deep in the pelvis, often heterogeneous in receptor expression (PSMA, CD44) and frequently hypoxic conditions that blunt photosensitizer (PS) delivery and limit ROS yield. Organic polymeric nanoparticles address these constraints by: (i) solubilizing/retaining PS, (ii) enabling molecular targeting, (iii) carrying oxygen or generating it *in situ*, and (iv) combining PDT with complementary modalities (PTT/US imaging) while preserving biocompatibility. The latest field review frames these strategies around polymer chemistry and nanoconstruction choices that tailor photophysics, tumor selectivity, and immune consequences of PDT 60.

#### 2.4.3 PDT + Chemotherapy / Autophagy Modulation

Engineered nanoplatforms pair tumour-localized photochemistry with light-triggered release of immune agonists, transforming PDT from a damage signal into a programmable vaccine. A 2023 Nature-Communications-reported “photoactivatable nanoagonist” delivered NIR-triggered cytotoxicity in synchrony with controlled agonist release, orchestrating ICD, APC activation, and systemic antitumour immunity. This chemical choreography yielded superior tumour control and memory versus either PDT or agonist alone, offering a blueprint for modular, light-addressable ISV 59. Although synergy between PDT and checkpoint blockade is well-demonstrated in syngeneic mouse models, translation to human tumors, particularly those with intrinsically low immunogenicity, such as glioblastoma or prostate cancer may require patient stratification based on immunogenicity markers, including tumor mutational burden (TMB) or PD-L1 expression, to better identify individuals likely to benefit. PDT remodels tumour immunogenicity and improves response to ICB, especially when coupled to physical or microenvironmental “priming” that enhances drug/light penetration again consistent with the vaccine-like logic of local antigen release plus adjuvancy 61.

Okada *et al.* evaluated the synergy between near-infrared photoimmunotherapy (NIR-PIT) and an intratumoral *in situ* vaccine (ISV) in a pancreatic cancer model, a tumor type known for its profound immunosuppressive properties. The researchers used an anti-CD44 antibody conjugated with IR700 dye for PIT, combined with the TLR9 agonist K3-SPG as the vaccine adjuvant. This combinatorial regimen resulted in a significant upregulation of interferon-related genes within the tumor, enhanced dendritic cell maturation, and elicited a potent CD8⁺ T-cell response. Functionally, it achieved both local tumor eradication and systemic immune protection, including abscopal effects on distant lesions. The antitumor efficacy was abrogated in CD8-depleted mice, confirming T-cell dependency. Moreover, the therapy induced long-term immunological memory that protected against tumor rechallenge. This work provides compelling evidence that PIT, when paired with innate immune adjuvants, can act as an effective *in situ* cancer vaccination strategy 62.

Sasaki *et al.* investigated the combination of talaporfin sodium-based PDT (TS-PDT) with anti-PD-1 ICB in syngeneic tumor models. TS-PDT was shown to induce classical features of ICD, including the exposure of calreticulin, ATP release, and HMGB1 liberation. When combined with anti-PD-1 antibody treatment, the regimen produced significantly enhanced tumor regression compared with either monotherapy. Mechanistic analyses confirmed that PDT not only provided direct tumor cytotoxicity but also created a pro-inflammatory milieu that primed T-cells for checkpoint inhibition. The combined approach elicited abscopal suppression of distant tumors and generated memory responses, suggesting that TS-PDT can serve as a clinically tractable ICD inducer to augment checkpoint blockade therapies. This study represents a key milestone, bridging PDT-induced ICD with systemic immunotherapy in a translationally relevant setting 63.

Equally critical is the immunological dimension. While PDT-induced ICD has catalyzed enthusiasm for PDT-immunotherapy combinations, only limited early-phase clinical trials have validated these synergistic concepts. Checkpoint inhibitor combinations and *in situ* vaccine strategies are mechanistically strong, but dosing schedules, patient selection, and immune-related adverse effects remain unresolved. Without standardized protocols and predictive biomarkers, clinical reproducibility will remain elusive. Taken together, the future of PDT will depend less on incremental advances in photosensitizers and more on resolving these translational barriers. Emphasis must shift toward harmonized dosimetry, rigorous toxicology of nanoplatforms, and carefully designed clinical trials that integrate PDT with precision oncology frameworks. Only by addressing these unresolved controversies can PDT progress from preclinical promise to clinical mainstream as a durable pillar of integrative cancer therapy.

## 3. Current Challenges

### 3.1. Limited Light Penetration

A primary limitation of conventional PDT lies in the restricted penetration depth of visible and NIR light in biological tissues, typically only few millimeters. This constraint renders PDT most effective for superficial or endoscopically accessible tumors, while deep-seated malignancies such as pancreatic, hepatic, and brain cancers remain difficult to treat.

Recent advancements such as two-photon excitation, UCNPs, and X-ray-induced scintillator-assisted PDT (X-PDT) have shown great promise in overcoming this issue. In particular, X-PDT utilizes deeply penetrating X-rays to activate scintillating nanoparticles that subsequently transfer energy to photosensitizers, generating ROS *in situ*. Similarly, Cherenkov radiation-induced PDT (CR-PDT) leverages the intrinsic luminescence emitted during high-energy radiation therapy to activate photosensitizers without external light sources.

Although these modalities have demonstrated efficacy in preclinical models, future research must focus on optimizing energy transfer efficiency, minimizing radiation dose, and improving biocompatibility of scintillators. The integration of image-guided radiotherapy systems with X-PDT and CR-PDT could further enhance spatial precision and safety, facilitating clinical translation.

### 3.2. Tumor Hypoxia

Hypoxia within the TME significantly reduces the efficacy of oxygen-dependent ROS generation in PDT, weakening both direct cytotoxic and immunogenic effects. Although approaches such as oxygen-generating nanocarriers, perfluorocarbon-based oxygen delivery, and hypoxia-activated prodrugs have been developed, their translation faces challenges related to biosafety and large-scale production.

Future prospects involve combining X-PDT or CR-PDT with oxygen-independent therapeutic mechanisms, such as type I PDT, radiodynamic therapy, or catalytic nanomedicine, to sustain ROS generation under hypoxia. Moreover, developing smart nanoplatforms that couple oxygen generation with X-ray activation may enable a synergistic effect, improving therapeutic outcomes in hypoxic tumors.

### 3.3. Tumor Microenvironment (TME) Barriers

The heterogeneous and immunosuppressive nature of the TME hinders photosensitizer accumulation and limits PDT-induced antitumor immunity. Aberrant vasculature, acidic pH, and high interstitial pressure further restrict effective drug delivery.

Emerging research on X-PDT and CR-PDT indicates that these approaches may partially alleviate such barriers by promoting localized oxidative stress, vascular normalization, and immune activation through deep-tissue energy deposition. Future strategies could integrate these modalities with TME-modulating agents or immune checkpoint inhibitors to achieve durable and systemic antitumor responses. Multi-modal theranostic platforms combining X-ray imaging, radiotherapy, and PDT may also provide real-time monitoring and adaptive control over treatment efficacy.

### 3.4. Photosensitizer Limitations

Despite progress in photosensitizer development, many current compounds suffer from poor water solubility, aggregation, and non-specific biodistribution, leading to prolonged skin photosensitivity. To enhance clinical feasibility, future efforts should focus on designing X-ray-responsive or Cherenkov-activated photosensitizers with high radioluminescence coupling efficiency and minimal dark toxicity. The integration of MOFs, scintillator-photosensitizer conjugates, and biodegradable nanocomposites could improve pharmacokinetics, tumor targeting, and activation selectivity. Furthermore, advances in computational modeling and AI-driven molecular design may accelerate the discovery of next-generation sensitizers tailored for X-PDT and CR-PDT.

### 3.5. Translational and Clinical Challenges

The translation of PDT-based modalities to clinical settings remains hindered by the lack of standardized light or radiation dosimetry, limited large-scale clinical trials, and high costs of advanced photosensitizers and delivery systems. For X-PDT and CR-PDT, additional challenges arise from radiation safety, dose optimization, and regulatory classification whether as radiotherapy or phototherapy.

To overcome these hurdles, future directions should prioritize standardized treatment protocols, interdisciplinary collaborations between radiation oncologists and photochemists, and integration of imaging-based dosimetry tools. Establishing multicenter clinical trials and developing cost-effective nanoscintillator formulations will be critical steps toward realizing the full therapeutic potential of these next-generation PDT modalities.

Recent technological innovations, including miniaturized implantable light-emitting diodes (LEDs) and the repurposing of existing clinical laser systems, are emerging as practical strategies to reduce device-related costs and infrastructure demands. These developments may facilitate broader clinical access to PDT, particularly in resource-limited or decentralized healthcare settings.

## 4. Opportunities Ahead

### 4.1. Nanotechnology Integration

Nanotechnology has opened new opportunities for enhancing PDT by enabling the precise delivery of photosensitizers, oxygen carriers, and immune adjuvants within a single platform. Smart nanocarriers with stimuli-responsive properties allow tumor-selective activation while minimizing off-target effects. These advances not only improve therapeutic efficacy but also broaden the scope of PDT in combination with other treatment modalities.

### 4.2. Immunotherapy Synergy

The recognition of PDT as a potent inducer of ICD provides an opportunity to position it as an “*in situ* cancer vaccine.” PDT can convert immunologically “cold” tumors into “hot” tumors, thereby sensitizing them to immune checkpoint inhibitors and other immunotherapies **10,51**. The combination of PDT with ICB, cancer vaccines, or adoptive T-cell therapies holds immense potential to achieve durable systemic responses against both primary and metastatic tumors.

### 4.3. Next-Generation Light Sources

Novel light-activation strategies, including bioluminescence resonance energy transfer (BRET), Cerenkov radiation, and implantable optical fiber devices, offer the potential to bypass the conventional limitation of shallow tissue penetration. These approaches can extend the applicability of PDT to deep-seated tumors while preserving spatial precision and minimizing collateral damage.

### 4.4. Precision Oncology and Image-Guided PDT

The integration of PDT with precision oncology frameworks and advanced imaging modalities represents another significant opportunity. Image-guided PDT enables real-time monitoring of photosensitizer distribution, treatment planning, and adaptive therapy adjustments. Such advances pave the way for highly personalized and minimally invasive PDT interventions that can be tailored to individual patient profiles.

### 4.5. Multidisciplinary Convergence

Finally, PDT sits at the crossroads of chemistry, materials science, photonics, and immunology. The convergence of these disciplines is driving the development of multifunctional platforms that combine therapeutic efficacy with diagnostic precision. As these interdisciplinary innovations continue to mature, PDT has the potential to evolve from an adjunctive modality into a central component of comprehensive cancer management strategies in the coming decade.

## 5. Future Prospects

Taken together, the future of PDT lies in its transformation from a light-dependent local therapy into a central pillar of integrative oncology. By leveraging nanomedicine, immunotherapy, and precision medicine, PDT has the potential not only to improve cancer survival outcomes but also to reshape the therapeutic landscape fundamentally.

### 5.1. Clinical Translation of Multifunctional Nanoplatforms

The next decade will demand the clinical translation of smart nanocarriers that have shown superior preclinical performance in delivering photosensitizers, oxygen carriers, and immune adjuvants. Large-scale, GMP-compliant manufacturing, detailed pharmacokinetics, and long-term biosafety studies will be essential. Particular attention should be paid to nanocarriers such as plasmonic gold cores, which may accumulate in reticuloendothelial organs, and MnO₂ nanoflowers, which require long-term studies to assess Mn²⁺ ion release and hepatic accumulation 16. Future PDT systems are expected to integrate therapeutic, diagnostic, and monitoring functions within single theranostic platforms, enabling precision treatment and real-time response assessment.

### 5.2. PDT as an Immunomodulatory Therapy

The ability of PDT to induce ICD positions it as a powerful immunomodulatory partner in cancer treatment. Clinical trials combining PDT with immune checkpoint inhibitors, CAR-T cells, and neoantigen-based vaccines could establish PDT as an *in situ* cancer vaccine, transforming it from a local therapy into a systemic treatment strategy against primary and metastatic disease.

### 5.3. Next-Generation Light Activation Strategies

Overcoming shallow tissue penetration will be critical for expanding PDT applications. BRET, Cerenkov radiation, X-ray-activated scintillators, and self-illuminating nanoparticles represent next-generation strategies that can deliver PDT to deep-seated tumors with high precision and minimal collateral damage.

### 5.4. Personalization through Precision Oncology

Precision oncology frameworks will increasingly guide future PDT approaches. Integration of genomic, proteomic, and imaging biomarkers will enable the selection of optimal photosensitizers, nanocarriers, and light dosimetry tailored to individual patients. Such personalized treatment strategies will maximize therapeutic efficacy while reducing off-target effects.

### 5.5. Interdisciplinary and Translational Collaboration

The evolution of PDT will rely heavily on multidisciplinary innovation. Advances in chemistry, nanotechnology, photonics, artificial intelligence, and tumor immunology will converge to design clinically relevant PDT platforms. Simultaneously, regulatory harmonization, cost-effective production, and multicenter randomized trials will be crucial for transitioning these innovations from the bench to the bedside.

### 5.6. Perspectives: Major Trends, Knowledge Gaps, and Future Directions

Despite the rapid evolution of PDT technologies, several overarching trends and research patterns are evident across recent literature. One notable trend is the shift from single-function photosensitizers toward multifunctional, stimuli-responsive nanoplatforms that integrate imaging, targeting, oxygen modulation, and combined PDT/PTT effects. Another emerging direction is the increasing adoption of oxygen-independent or oxygen-generating systems, which reflects an attempt to solve the persistent challenge of tumor hypoxia. Additionally, PDT is gradually transitioning from a local cytotoxic therapy to a systemic immunomodulatory strategy, with growing emphasis on combining PDT-induced ICD with immune-checkpoint blockade or vaccine-like platforms.

Despite these advances, important knowledge gaps remain. First, heterogeneous intratumoral oxygen distribution still limits reproducibility of PDT efficacy, even in studies using oxygen-generating nanomaterials. Second, photosensitizer pharmacokinetics and biodistribution remain poorly understood, particularly in human tumors, where stromal density and vascular abnormalities vary widely. Third, many multifunctional nanoplatforms suffer from overcomplexity, weak scalability, and unclear long-term biosafety, making clinical translation challenging. Finally, while photoimmunotherapy is promising, the interplay between ROS signaling and immune pathways remains insufficiently characterized.

Future research must emphasize mechanistically guided design, clinical-grade manufacturing, and improved tumor models that better mimic human pathology. Robust long-term biosafety and clearance studies, standardized light-dosing protocols, and real-time monitoring tools are urgently needed to ensure translational viability. The integration of PDT with immunotherapy, gene regulation, and artificial intelligence-driven light planning may represent the next transformative steps. By addressing these gaps, the field can move toward predictable, personalized, and clinically effective PDT strategies.

## 6. Conclusion

PDT has matured from a localized light-dependent cytotoxic modality into a versatile platform that integrates nanotechnology, immunology, and precision medicine. Over the past decade, innovations in photosensitizer design, nanocarrier engineering, and light-activation strategies have substantially broadened the scope of PDT. Importantly, PDT is now increasingly recognized as an immunomodulatory partner capable of synergizing with ICB, cancer vaccines, and photothermal therapy to achieve durable systemic effects. Yet, despite this impressive scientific progress, the clinical translation of PDT remains cautious. Light penetration, tumor hypoxia, and heterogeneous tumor biology continue to restrict efficacy in real-world oncology. Most multifunctional nanoplatforms though powerful in preclinical studies still face unresolved concerns regarding biosafety, reproducibility, and regulatory approval. Likewise, while PDT-induced ICD provides a compelling rationale for combination immunotherapies, rigorous clinical evidence remains sparse, and the risk of immune-related toxicities requires careful management. Looking forward, the next decade of PDT will depend on resolving these translational bottlenecks. Priority areas include standardized light-dosimetry protocols, harmonized nanoplatform toxicology, and biomarker-driven patient selection for PDT-immunotherapy trials. Equally critical will be the interdisciplinary convergence of materials science, photonics, oncology, and immunology to design clinically tractable systems that combine efficacy with safety. By directly confronting these challenges, PDT can progress from an experimental adjunct to a central component of integrative oncology, thereby reshaping the therapeutic landscape for both primary and metastatic cancers.

## Figures and Tables

**Table 1:** Comparative Strategies in PDT

Strategy	Key Mechanism	Advantages	Limitations	Technology Readiness Level (TRL)	Clinical Outlook
Activatable PS (pH/enzyme/redox sensitive)	Tumor microenvironment triggers “ON” state	High selectivity, reduced systemic toxicity	Variable activation efficiency, heterogeneity across tumors	Preclinical (TRL 3-4)	Promising for superficial cancers; clinical translation needs controlled activation proof
Bioluminescent PDT (BL-PDT)	Internal bioluminescence activates PS	Bypasses light penetration limit; effective in deep tumors	Complexity of bioluminescent reaction; enzyme stability and energy yield limitations	Early preclinical (TRL 2-3)	Promising deep-tissue strategy; however, clinical translation remains distant due to challenges in sustaining efficient *in vivo* bioluminescent reactions, low photon flux for PS activation, and potential immunogenicity of luciferase-based systems.
Red/NIR PS (phthalocyanines, indomethacin-conjugates)	NIR absorption for deep penetration	Better tissue penetration, reduced collateral damage	Aggregation, limited tumor selectivity	Clinical (TRL 6-7, some in trials)	Closest to translation; improved scaffolds may enhance targeting
Upconversion nanoparticles (UCNPs)	Convert NIR → visible light	Enables deep-tissue PDT, tunable emission	Low quantum yield, long-term toxicity unknown	Preclinical (TRL 3-4)	Attractive for focal therapy; regulatory hurdles
TSPO-targeted PS	Mitochondria-localized PS	High ROS yield, mitochondrial apoptosis	Need tumor-specific TSPO overexpression	Preclinical (TRL 3-4)	Good candidate for precision oncology; biomarker validation required
Pt(IV) O₂-independent PS	Direct biomolecule oxidation	Effective in hypoxia, bypasses ROS limitation	Novel chemistry, safety untested	Preclinical (TRL 2-3)	High potential for hypoxia-resistant tumors; long path to clinic
Nanocarriers (liposomes, hybrid NPs, porphysomes)	Improved solubility, EPR targeting	Enhanced accumulation, multimodal imaging	Scale-up & reproducibility issues	Mid-to-late preclinical (TRL 4-5)	Liposomes are precedent (Doxil®); porphysomes promising
Stimuli-responsive nanoinhibitors	Dual action: ROS + pathway inhibition	Overcomes resistance, synergistic	Complexity of design, biosafety concerns	Preclinical (TRL 3-4)	Futuristic, but complex for regulatory approval
Hypoxia-relief platforms (MnO₂, O₂ nanoemulsions, microneedle patches)	Generate/supply O₂ at the tumor site	Directly improves PDT efficacy in hypoxic tumors	Short-term O₂ supply, stability issues	Preclinical (TRL 3-4)	Strong translational promise, esp. microneedle patches
PDT + Immunotherapy	PDT-induced ICD + checkpoint blockade	Durable systemic immunity, abscopal effects	Complex dosing, immune-related adverse effects	Preclinical to early clinical (TRL 5-6)	Strong candidate for next-gen clinical trials

## Abbreviations

ABCG2: ATP-binding cassette subfamily G member 2
ALA: 5-Aminolevulinic Acid
APC: Antigen-Presenting Cell
BRET: Bioluminescence Resonance Energy Transfer
COX: Cyclooxygenase
CTL: Cytotoxic T Lymphocyte
DC: Dendritic Cell
DNA: Deoxyribonucleic Acid
ER: Endoplasmic Reticulum
GMP: Good Manufacturing Practice
GSH: Glutathione
ICB: Immune Checkpoint Blockade
ICD: Immunogenic Cell Death
IR700: Near-infrared dye IR700
MAPK: Mitogen-Activated Protein Kinase
MOF: Metal-Organic Framework
MRI: Magnetic Resonance Imaging
NIR: Near-Infrared
NP: Nanoparticle
NSAID: Non-Steroidal Anti-Inflammatory Drug
OPNP: Organic-Inorganic Photodynamic Nanoplatform
PDA: Polydopamine
PDT: Photodynamic Therapy
PpIX: Protoporphyrin IX
PS: Photosensitizer
PSMA: Prostate-Specific Membrane Antigen
PVP: Polyvinylpyrrolidone
ROS: Reactive Oxygen Species
TME: Tumor Microenvironment
TLR: Toll-Like Receptor
TLR7: Toll-Like Receptor 7
UCNP: Upconversion Nanoparticle
US: Ultrasound

## References

[1] Song M, Yoon H, Yoon H, Lee HM, Chae YJ, Chang JE (2024). Enhanced anticancer efficacy of photodynamic therapy in combination with immunotherapy. J Photochem Photobiol B.

[2] Ali W, Kulsoom, Wang F (2024). Molecular probes for monitoring pyroptosis: design, imaging and theranostic application. Apoptosis.

[3] Tian J, Li B, Zhang F, Yao Z, Song W, Tang Y (2023). Activatable Type I Photosensitizer with Quenched Photosensitization Pre and Post Photodynamic Therapy. Angew Chem Int Ed Engl.

[4] Tang Y, Wang X, Zhu G, Liu Z, Chen X-M, Bisoyi HK (2023). Hypoxia-Responsive Photosensitizer Targeting Dual Organelles for Photodynamic Therapy of Tumors. Small.

[5] Kulsoom Ali W, Wang F (2025). Advancement in synthetic gene circuits engineering: An alternative strategy for microRNA imaging and disease theranostics. Biotechnol Adv.

[6] Deng C, Zhang J, Hu F, Han S, Zheng M, An F (2024). A GSH-Responsive Prodrug with Simultaneous Triple-Activation Capacity for Photodynamic/Sonodynamic Combination Therapy with Inhibited Skin Phototoxicity. Small.

[7] Yan Y, Kulsoom, Sun Y, Li Y, Wang Z, Xue L (2025). Advancing cancer therapy: Nanomaterial-based encapsulation strategies for enhanced delivery and efficacy of curcumin. Mater Today Bio.

[8] Deng C, Zhang J, Yang Y, Ding Y, An F, Wang F (2025). Chemodynamic Therapy Enhanced (131)I-Radiotherapy for Efficient Inhibition on Cancer Growth and Metastasis. Small.

[9] Yang Z, Qin R, Ruan D, Hu C, Li W, Zhou J (2025). Ce6-DNAzyme-Loaded Metal-Organic Framework Theranostic Agents for Boosting miRNA Imaging-Guided Photodynamic Therapy in Breast Cancer. ACS Nano.

[10] Ji B, Wei M, Yang B (2022). Recent advances in nanomedicines for photodynamic therapy (PDT)-driven cancer immunotherapy. Theranostics.

[11] Turubanova VD, Balalaeva IV, Mishchenko TA, Catanzaro E, Alzeibak R, Peskova NN (2019). Immunogenic cell death induced by a new photodynamic therapy based on photosens and photodithazine. J Immunother Cancer.

[12] Yi G, Hong SH, Son J, Yoo J, Park C, Choi Y (2018). Recent advances in nanoparticle carriers for photodynamic therapy. Quantitative Imaging in Medicine and Surgery.

[13] Su M, Chen Y, Jia L, Zhang Z (2022). Camptothecin-Loaded and Manganese Dioxide-Coated Polydopamine Nanomedicine Used for Magnetic Resonance Imaging Diagnosis and Chemo-Photothermal Therapy for Lung Cancer. Int J Nanomedicine.

[14] Kan D, Ding R, Yang H, Jia Y, Lei K, Wang Z (2025). Synergistic strategies in photodynamic combination therapy for cancer: mechanisms, nanotechnology, and clinical translation. Frontiers in Oncology. 2025; Volume 15 -.

[15] Cui S, Yin D, Chen Y, Di Y, Chen H, Ma Y (2013). In vivo targeted deep-tissue photodynamic therapy based on near-infrared light triggered upconversion nanoconstruct. ACS Nano.

[16] Li X, Zheng BY, Ke MR, Zhang Y, Huang JD, Yoon J (2017). A Tumor-pH-Responsive Supramolecular Photosensitizer for Activatable Photodynamic Therapy with Minimal In Vivo Skin Phototoxicity. Theranostics.

[17] Li Y, He G, Fu L-H, Younis MR, He T, Chen Y (2022). A microneedle patch with self-oxygenation and glutathione depletion for repeatable photodynamic therapy. ACS nano.

[18] Tang Y, Li Y, Li B, Song W, Qi G, Tian J (2024). Oxygen-independent organic photosensitizer with ultralow-power NIR photoexcitation for tumor-specific photodynamic therapy. Nat Commun.

[19] Li J, Zhang Q, Yang H, Lu W, Fu Y, Xiong Y (2024). Sequential dual-locking strategy using photoactivated Pt(IV)-based metallo-nano prodrug for enhanced chemotherapy and photodynamic efficacy by triggering ferroptosis and macrophage polarization. Acta pharmaceutica Sinica B.

[20] Xie Q, Su M, Liu Y, Zhang D, Li Z, Bai M (2021). Translocator protein (TSPO)-Targeted agents for photodynamic therapy of cancer. Photodiagnosis and photodynamic therapy.

[21] Broekgaarden M, Weijer R, van Gulik TM, Hamblin MR, Heger M (2015). Tumor cell survival pathways activated by photodynamic therapy: a molecular basis for pharmacological inhibition strategies. Cancer Metastasis Rev.

[22] Mejdahl Nielsen M, Mathiesen S, Suominen A, Sørensen K, Ifversen M, Mølgaard C (2021). Altered body composition in male long-term survivors of paediatric allogeneic haematopoietic stem cell transplantation: impact of conditioning regimen, chronic graft-versus-host disease and hypogonadism. Bone Marrow Transplant.

[23] Sun W, Yu H, Wang D, Li Y, Tian B, Zhu S (2021). MnO(2) nanoflowers as a multifunctional nano-platform for enhanced photothermal/photodynamic therapy and MR imaging. Biomater Sci.

[24] Levi V (2022). Seeing the smallest rotary biomotor. Nat Rev Mol Cell Biol.

[25] Kuzmina NS, Fedotova EA, Jankovic P, Gribova GP, Nyuchev AV, Fedorov AY (2024). Enhancing Precision in Photodynamic Therapy: Innovations in Light-Driven and Bioorthogonal Activation. Pharmaceutics.

[26] Li C, Wang P, Wang D, Shi L, Zhou Z, Zhang L (2022). Fluorescence kinetics study of twice laser irradiation based HpD-PDT for nonmelanoma skin cancer. Lasers Surg Med.

[27] Parthiban V, Yen PYM, Uruma Y, Lai P-S (2020). Designing Synthetic Glycosylated Photosensitizers for Photodynamic Therapy. Bulletin of the Chemical Society of Japan.

[28] Bhaumik J, Mittal AK, Banerjee A, Chisti Y, Banerjee UC (2015). Applications of phototheranostic nanoagents in photodynamic therapy. Nano Research.

[29] O'Mahoney P, Samuel ID, Eadie E, Ibbotson S (2021). Fluorescence and thermal imaging of non-melanoma skin cancers before and during photodynamic therapy. Photodiagnosis and Photodynamic Therapy.

[30] Yan H, Forward S, Kim K-H, Wu Y, Hui J, Kashiparekh A (2023). All-natural-molecule, bioluminescent photodynamic therapy results in complete tumor regression and prevents metastasis. Biomaterials.

[31] Chang Y, Li X, Zhang L, Xia L, Liu X, Li C (2017). Precise Photodynamic Therapy of Cancer via Subcellular Dynamic Tracing of Dual-loaded Upconversion Nanophotosensitizers. Sci Rep.

[32] Fu L, Huang Y, Hou J, Sun M, Wang L, Wang X (2022). A Raman/fluorescence dual-modal imaging guided synergistic photothermal and photodynamic therapy nanoplatform for precision cancer theranostics. J Mater Chem B.

[33] Siriwibool S, Wangngae S, Chansaenpak K, Wet-Osot S, Lai RY, Noisa P (2022). Indomethacin-based near-infrared photosensitizer for targeted photodynamic cancer therapy. Bioorg Chem.

[34] Deng Z, Li H, Chen S, Wang N, Liu G, Liu D (2023). Near-infrared-activated anticancer platinum (IV) complexes directly photooxidize biomolecules in an oxygen-independent manner. Nature Chemistry.

[35] Liu J, Li Y, Wang F (2025). Shedding light on Alzheimer's disease: Recent advances in highly selective fluorescent probes. Coordination Chemistry Reviews.

[36] Udrea AM, Smarandache A, Dinache A, Mares C, Nistorescu S, Avram S (2023). Photosensitizers-Loaded Nanocarriers for Enhancement of Photodynamic Therapy in Melanoma Treatment. Pharmaceutics.

[37] Wang P, Tang H, Zhang P (2016). Plasmonic Nanoparticle-based Hybrid Photosensitizers with Broadened Excitation Profile for Photodynamic Therapy of Cancer Cells. Sci Rep.

[38] Silva LB, Castro KADF, Botteon CEA, Oliveira CLP, da Silva RS, Marcato PD (2021). Hybrid Nanoparticles as an Efficient Porphyrin Delivery System for Cancer Cells to Enhance Photodynamic Therapy. Frontiers in Bioengineering and Biotechnology. 2021; Volume 9 -.

[39] Silva LB, Castro KA, Botteon CE, Oliveira CL, Da Silva RS, Marcato PD (2021). Hybrid nanoparticles as an efficient porphyrin delivery system for cancer cells to enhance photodynamic therapy. Frontiers in Bioengineering and Biotechnology.

[40] Guidolin K, Ding L, Chen J, Wilson BC, Zheng G (2021). Porphyrin-lipid nanovesicles (Porphysomes) are effective photosensitizers for photodynamic therapy. Nanophotonics.

[41] Jiang Z, Zhang J, Jin J, Zhang X, Kadeerhan G, Guo H (2025). Enhanced NIR-II Nanoparticle Probe for PSMA-Targeted Molecular Imaging and Prostate Cancer Diagnosis. Int J Nanomedicine.

[42] Ngen EJ, Chen Y, Azad BB, Boinapally S, Jacob D, Lisok A (2021). Prostate-specific membrane antigen (PSMA)-targeted photodynamic therapy enhances the delivery of PSMA-targeted magnetic nanoparticles to PSMA-expressing prostate tumors. Nanotheranostics.

[43] Dai L, Shen G, Wang Y, Yang P, Wang H, Liu Z (2021). PSMA-targeted melanin-like nanoparticles as a multifunctional nanoplatform for prostate cancer theranostics. Journal of Materials Chemistry B.

[44] Lai HW, Tani Y, Sukatta U, Rugthaworn P, Thepyos A, Yamamoto S (2023). Mangostin enhances efficacy of aminolevulinic acid-photodynamic therapy against cancer through inhibition of ABCG2 activity. Photodiagnosis and Photodynamic Therapy.

[45] Jiang Y, Li J, Zeng Z, Xie C, Lyu Y, Pu K (2019). Organic photodynamic nanoinhibitor for synergistic cancer therapy. Angewandte Chemie International Edition.

[46] Wang Y, Huo J, Li S, Huang R, Fan D, Cheng H (2022). Self-rectifiable and hypoxia-assisted chemo-photodynamic nanoinhibitor for synergistic cancer therapy. ACS Applied Materials & Interfaces.

[47] Hong L, Pliss AM, Zhan Y, Zheng W, Xia J, Liu L (2020). Perfluoropolyether Nanoemulsion Encapsulating Chlorin e6 for Sonodynamic and Photodynamic Therapy of Hypoxic Tumor. Nanomaterials (Basel).

[48] Deng Y, Zhang Q, Liu G, Lin T, Zhang W, He X (2022). Self-Assembled PSMA-Targeted Nanoparticles Enhanced Photodynamic Therapy in Prostate Cancer. Journal of Nanomaterials.

[49] Hu J, Luo H, Qu Q, Liao X, Huang C, Chen J (2020). Cell Membrane-Inspired Polymeric Vesicles for Combined Photothermal and Photodynamic Prostate Cancer Therapy. ACS Appl Mater Interfaces.

[50] Zhang L, Ji Z, Zhang J, Yang S (2019). Photodynamic therapy enhances skin cancer chemotherapy effects through autophagy regulation. Photodiagnosis and photodynamic therapy.

[51] Li J, Pang E, An J, Xiong Z, Kim E, Lan M (2025). Off-Photosensitizing Derived Immuno-Photodynamic Therapy toward Postoperative Care. Advanced Functional Materials.

[52] Sun L, Wu H, Zhang Z, Wu K, Sun J, Dong X (2025). A Smart Visualized Phototherapy Switch: From NIR-I Imaging-Guided Photodynamic Therapy to NIR-II-Guided Photothermal Therapy for Enhanced Cascade Tumor Photoablation. Aggregate.

[53] Shafirstein G, Oakley E, Hamilton S, Habitzruther M, Chamberlain S, Sexton S (2022). In vivo models for studying interstitial photodynamic therapy of locally advanced cancer. Photodynamic Therapy: Methods and Protocols: Springer.

[54] Sasaki M, Tanaka M, Sasaki Y, Kojima Y, Suzuki T, Nishie H (2025). cGAS-STING Pathway Activation Enhances Antitumor Effect of Talaporfin Photodynamic Therapy Through ROS Production. Cancer Sci.

[55] Gurung P, Lim J, Shrestha R, Kim Y-W (2023). Chlorin e6-associated photodynamic therapy enhances abscopal antitumor effects via inhibition of PD-1/PD-L1 immune checkpoint. Scientific Reports.

[56] Tian J, Chen C, Du X, Wang M (2024). Near-infrared photoimmunotherapy in cancer treatment: a bibliometric and visual analysis. Frontiers in Pharmacology. 2024; Volume 15 -.

[57] Inagaki FF, Kano M, Furusawa A, Kato T, Okada R, Fukushima H (2024). Near-infrared photoimmunotherapy targeting PD-L1: Improved efficacy by preconditioning the tumor microenvironment. Cancer Science.

[58] Zheng ZY, Lin W, Su JW, Huang QF, Zhang C, Pan WX (2024). NIR-715 photodynamic therapy induces immunogenic cancer cell death by enhancing the endoplasmic reticulum stress response. Cell Death Dis.

[59] Wan J, Ren L, Li X, He S, Fu Y, Xu P (2023). Photoactivatable nanoagonists chemically programmed for pharmacokinetic tuning and in situ cancer vaccination. Proc Natl Acad Sci U S A.

[60] Ling J, Gu R, Liu L, Chu R, Wu J, Zhong R (2024). Versatile Design of Organic Polymeric Nanoparticles for Photodynamic Therapy of Prostate Cancer. ACS Mater Au.

[61] Lobo CS, Mendes MIP, Pereira DA, Gomes-da-Silva LC, Arnaut LG (2023). Photodynamic therapy changes tumour immunogenicity and promotes immune-checkpoint blockade response, particularly when combined with micromechanical priming. Scientific Reports.

[62] Yaku H, Takahashi K, Okada H, Kobiyama K, Shiokawa M, Uza N (2024). Near-infrared photoimmunotherapy as a complementary modality to in situ vaccine in a preclinical pancreatic cancer model. Biochem Biophys Res Commun.

[63] Sasaki M, Tanaka M, Kojima Y, Nishie H, Shimura T, Kubota E (2023). Anti-tumor immunity enhancement by photodynamic therapy with talaporfin sodium and anti-programmed death 1 antibody. Molecular Therapy-Oncolytics.

[64] Sasaki M, Tanaka M, Kojima Y, Nishie H, Shimura T, Kubota E (2023). Anti-tumor immunity enhancement by photodynamic therapy with talaporfin sodium and anti-programmed death 1 antibody. Mol Ther Oncolytics.

